# Colorless Polyimides Derived from 5,5′-bis(2,3-norbornanedicarboxylic anhydride): Strategies to Reduce the Linear Coefficients of Thermal Expansion and Improve the Film Toughness

**DOI:** 10.3390/polym15183838

**Published:** 2023-09-20

**Authors:** Masatoshi Hasegawa, Takuya Miyama, Junichi Ishii, Daisuke Watanabe, Akira Uchida

**Affiliations:** 1 Department of Chemistry, Faculty of Science, Toho University, 2-2-1 Miyama, Funabashi 274-8510, Chiba, Japan; 2 High Performance Materials Research & Development Department, High Performance Materials Company, ENEOS Corp., Yokohama 231-0815, Kanagawa, Japan; 3 Department of Biomolecular Science, Faculty of Science, Toho University, 2-2-1 Miyama, Funabashi 274-8510, Chiba, Japan

**Keywords:** colorless polyimides, 5,5′-bis(2,3-norbornanedicarboxylic anhydride), optical transparency, linear coefficients of thermal expansion (CTE), film toughness, solution processability, one-pot polycondensation

## Abstract

In this paper, novel colorless polyimides (PIs) derived from 5,5′-bis(2,3-norbornanedicarboxylic anhydride) (BNBDA) were presented. The results of single-crystal X-ray structural analysis using a BNBDA-based model compound suggested that it had a unique steric structure with high structural linearity. Therefore, BNBDA is expected to afford new colorless PI films with an extremely high glass transition temperature (*T*_g_) and a low linear coefficient of thermal expansion (CTE) when combined with aromatic diamines with rigid and linear structures (typically, 2,2′-bis(trifluoromethyl)benzidine (TFMB)). However, the polyaddition of BNBDA and TFMB did not form a PI precursor with a sufficiently high molecular weight; consequently, the formation of a flexible, free-standing PI film via the two-step process was inhibited because of its brittleness. One-pot polycondensation was also unsuccessful in this system because of precipitation during the reaction, probably owing to the poor solubility of the initially yielded BNBDA/TFMB imide oligomers. The combinations of (1) the structural modification of the BNBDA/TFMB system, (2) the application of a modified one-pot process, in which the conditions of the temperature-rising profile, solvents, azeotropic agent, catalysts, and reactor were refined, and (3) the optimization of the film preparation conditions overcame the trade-off between low CTE and high film toughness and afforded unprecedented PI films with well-balanced properties, simultaneously achieving excellent optical transparency, extremely high *T*_g_, sufficiently high thermal stability, low CTE, high toughness, relatively low water uptake, and excellent solution processability.

## 1. Introduction

Aromatic polyimides (PIs) are the most reliable, electrically insulating, and heat-resistant polymeric materials because of their extremely low contents of metallic, halogenic, and monomeric impurities, the highest class of fire retardancy and short-term heat resistance (extremely high glass transition temperatures, *T*_g_s) against solder-reflowing processes, resistance to various chemicals used in device fabrication processes, and excellent mechanical properties. Therefore, PIs have been primarily applied as electrical insulation films in a variety of electronic devices. Owing to their outstanding practical values, the chemistry, physics, characterization techniques, manufacturing processes, and applications of PIs have been extensively studied [1,2,3,4,5,6,7,8,9,10,11,12,13,14,15,16,17].

PI films can be produced via simple and clean processes using commercially available abundant monomers. The copolymerization approach using multiple monomers further enhances the tunability of structural modification. However, in recent years, the desired properties of PIs have diversified, and each requirement level has significantly increased. Therefore, it has become difficult to meet these demands by simply combining the existing monomers.

A recent urgent and challenging issue is the development of high-temperature, colorless polymeric materials that are practically useful for various potential applications such as the substrates in image display devices, touch sensors, transparent flexible printed circuit boards, flexible solar cells, and cover windows in foldable tablets and smart phones. Optically transparent (colorless) PIs are promising candidates for these optoelectronic applications. However, conventional wholly aromatic PI films usually exhibit intense coloration arising from intra- and intermolecular charge–transfer (CT) interactions [18]. Among wholly aromatic PIs, almost colorless PIs is virtually limited to a PI system derived from 4,4′-(hexafluoroisopropylidene)diphthalic anhydride (6FDA) and 2,2′-bis(trifluoromethyl)benzidine (TFMB). However, this PI film does not exhibit low thermal expansion property, which is indispensable for ensuring thermal dimensional stability [19,20].

**Figure 1:**
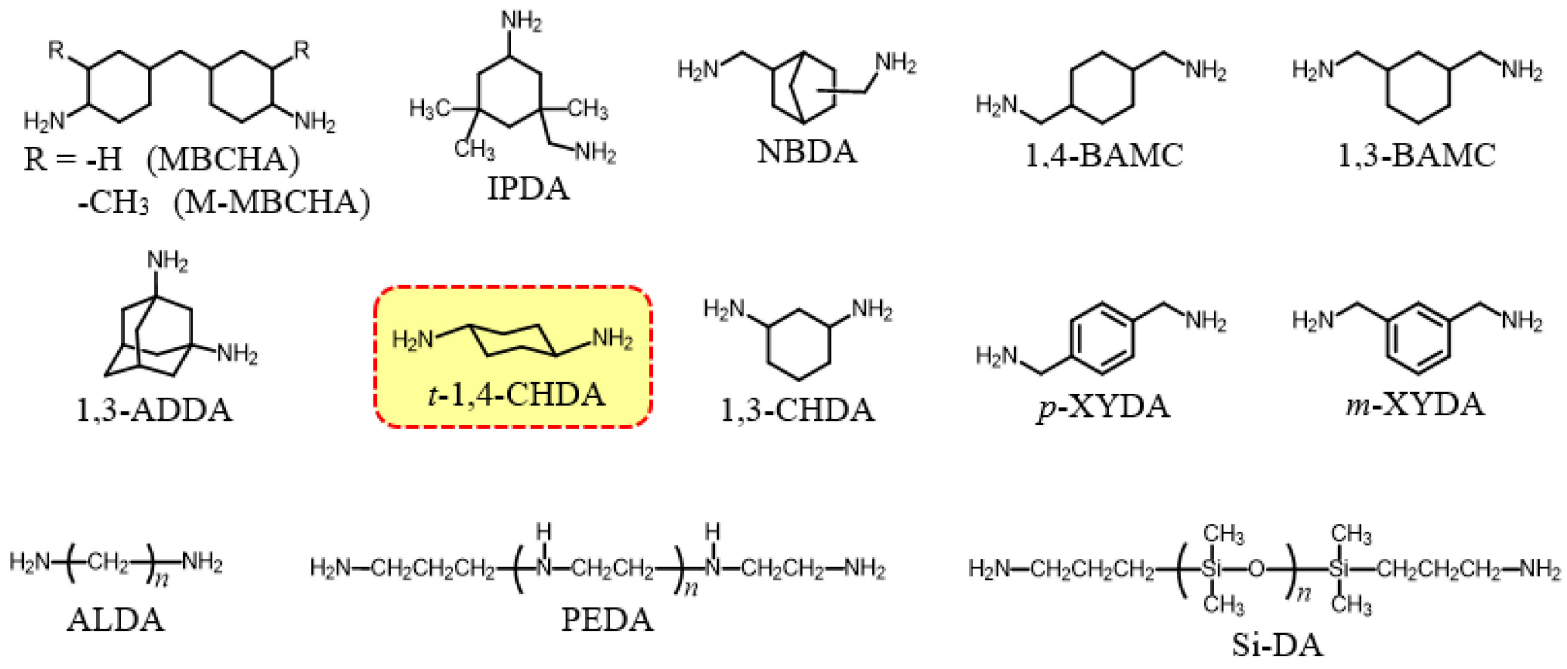
Structures and abbreviations of typical aliphatic diamines. The highlighted diamine represents that with the strongest salt formability.

The intense coloration of wholly aromatic PI films can be effectively eliminated by replacing either aromatic tetracarboxylic dianhydrides or aromatic diamines with aliphatic ones (usually cycloaliphatic (alicyclic) monomers) to impart heat resistance to the resultant PIs [18,21,22,23,24,25,26,27,28,29,30,31,32,33,34,35,36,37,38,39,40,41,42,43,44,45,46,47,48,49,50,51,52]. Figure 1 shows the molecular structures of the typical aliphatic diamine monomers.

**Figure 2:**
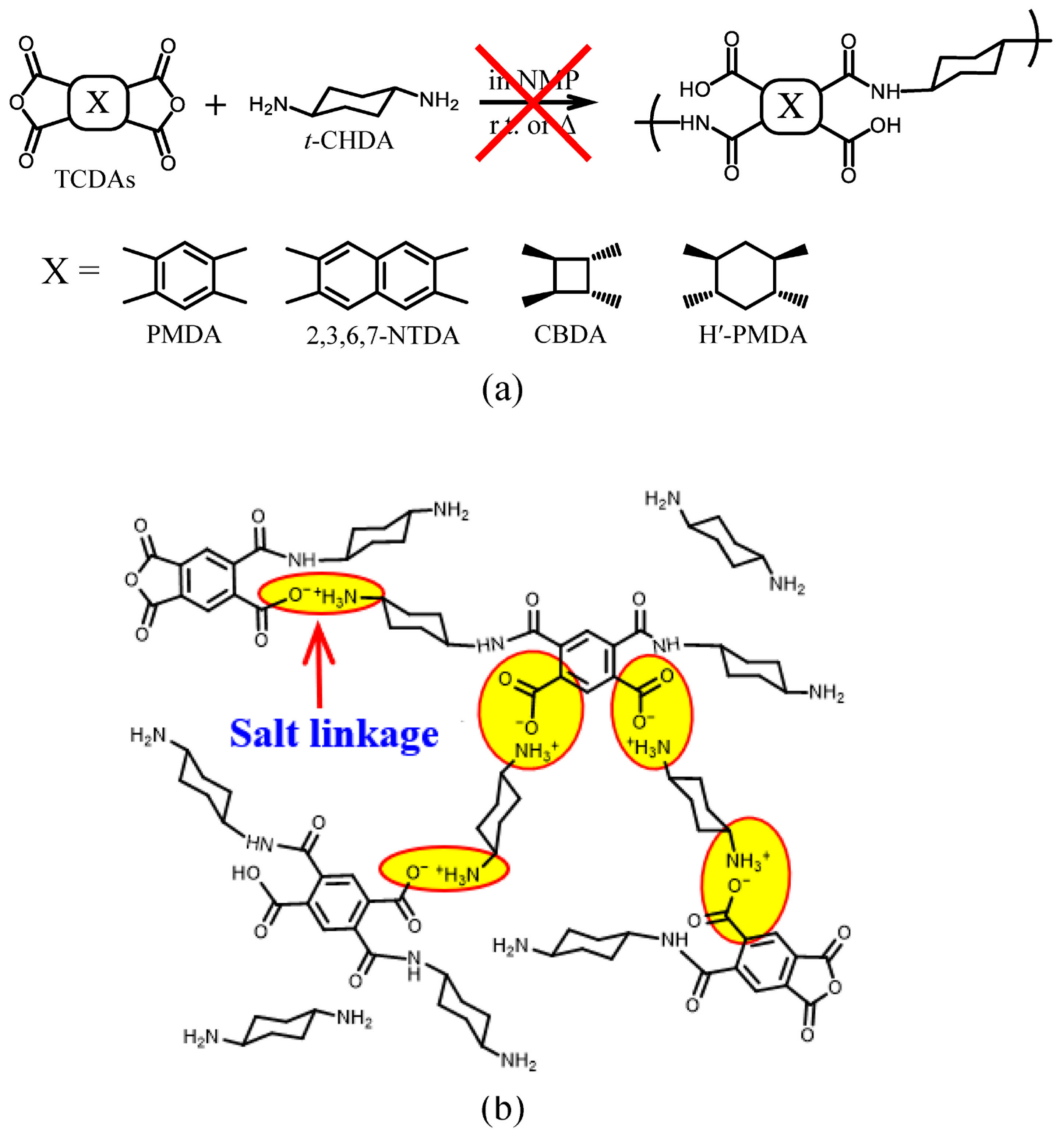
Typical systems where the equimolar polyaddition of *t*-CHDA and tetracarboxylic dianhydrides (TCDAs) is unsuccessful, owing to the complete insolubility of the salt formed in the initial reaction stage (**a**) and a schematic illustration of predicted crosslink structure based on salt formation in a typical system (PMDA/*t*-CHDA) (**b**).

However, the use of aliphatic diamines causes a serious problem, namely, the formation of poorly soluble salts between the COOH groups of the initially formed low-molecular-weight amic acids (AAs) and the unreacted NH_2_ groups of the added diamine and disturbs the smooth progress of polyaddition [38]. The poor solubility of the salts is likely related to its cross-linked structure, as schematically depicted in Figure 2. When non-rigid structures of aliphatic diamines (e.g., MBCHA, IPDA, and ALDA in Figure 1) were used, the initially formed salts gradually dissolve and participate in the polymerization during prolonged stirring at room temperature. In contrast, *trans*-1,4-cyclohexanediemine (*t*-CHDA) with a rigid structure tends to form a more “robust” salt. In particular, the combinations of *t*-CHDA and the rigid structures of tetracarboxylic dianhydrides (TCDAs, Figure 2 for typical examples) afford completely insoluble salts, which do not dissolve at all even after prolonged stirring, heating, and dilution [32,53,54].

**Figure 3:**
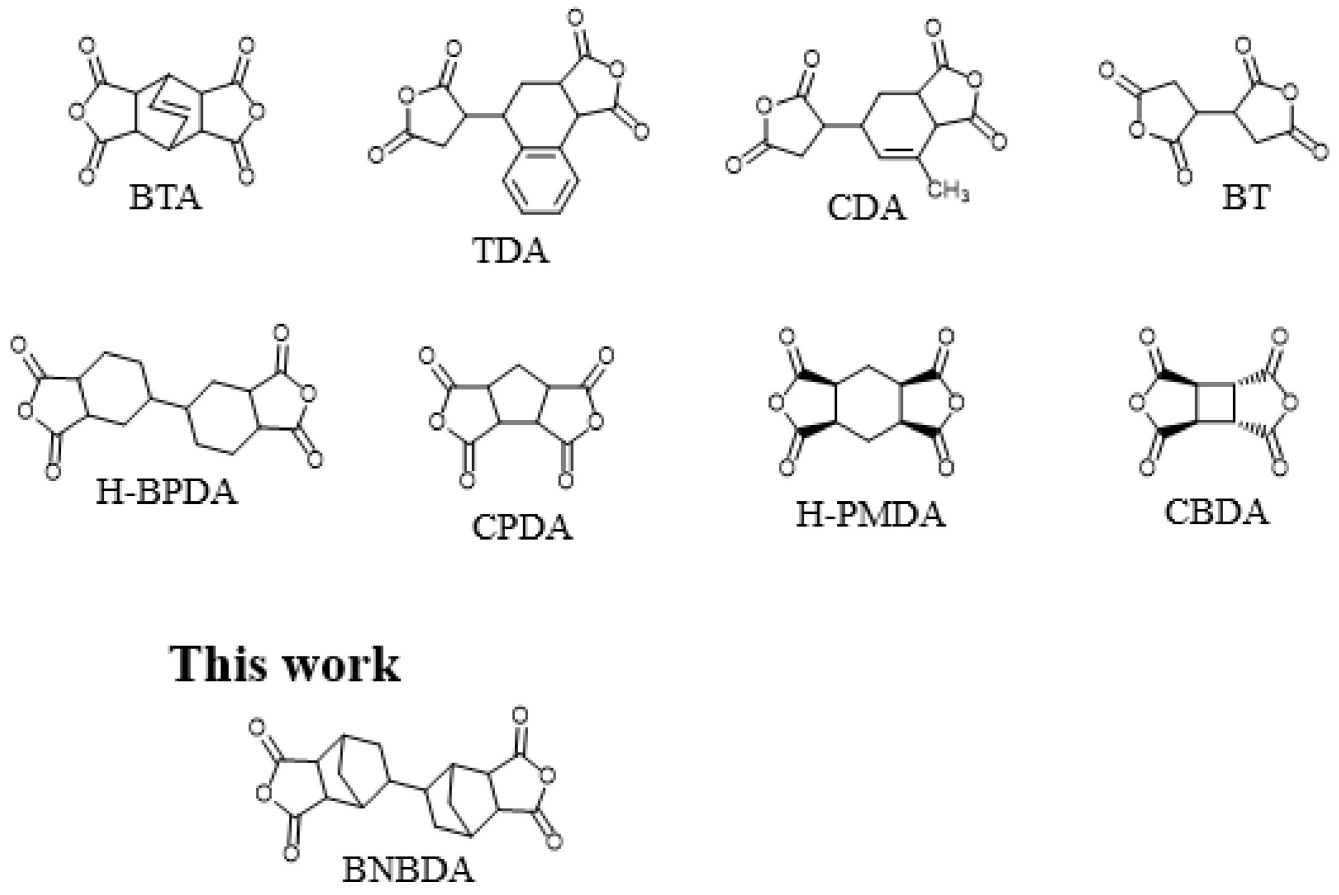
Structures and abbreviations of typical cycloaliphatic tetracarboxylic dianhydrides and 5,5′-bis(2,3-norbornanedicarboxylic anhydride) (BNBDA) used in this study.

In contrast, when cycloaliphatic TCDAs and aromatic diamines were combined, no salt formation occurs during the polyaddition. Therefore, this type of semi-aromatic colorless PI systems has been more extensively studied [26,27,28,29,30,31,32,33,34,35,36,37,38,39,40,41,42,43,44,45,46,47]. The structures of typical cycloaliphatic TCDAs are shown in Figure 3. However, commercially available cycloaliphatic TCDAs, that are acceptable in terms of their manufacturing costs, accessibility, and the heat resistance of the resultant PIs, are virtually limited to hydrogenated pyromellitic dianhydride (H-PMDA). However, H-PMDA has a crucial drawback, that is, insufficient polyaddition reactivity with specific aromatic diamines (typically, TFMB), which results in a very brittle PI film with cracks after the conventional two-step process [45]. In addition, even when H-PMDA was combined with rigid/linear structures of diamines, the resulting PI films are usually difficult to achieve low thermal expansion properties (specifically, low linear coefficients of thermal expansion (CTE)), owing to the non-linear/non-planar steric structure of the H-PMDA-based diimide (H-PMDI) units in the main chains [45].

In contrast, 1,2,3,4-cyclobutanetetracarboxylic dianhydride (CBDA) exhibits high polyaddition reactivity with TFMB, which is most favorable for enhancing the transparency and reducing the CTE, and affords a free-standing, colorless PI film with a very high *T*_g_ and low CTE via the conventional two-step process [55]. However, the use of CBDA is disadvantageous in terms of the film ductility [56] and solution processability [31] of the resulting PIs. In addition, it is difficult to significantly reduce the manufacturing cost of CBDA because it can be synthesized only via the photo-dimerization of maleic anhydride in solution [26,31], which is not suitable for large-scale production.

Hence, novel cycloaliphatic TCDAs that have high polymerization reactivity with TFMB and afford PI films while simultaneously achieving high optical transparency, high *T*_g_, low CTE, high toughness, and solution processability, have been strongly anticipated. In this study, novel colorless PIs derived from a cycloaliphatic TCDA, 5,5′-bis(2,3-norbornanedicarboxylic anhydride) (BNBDA, Figure 3) are proposed [57]. Moreover, the polymerization reactivity of BNBDA, film properties, and solution processability of a series of the resultant BNBDA-based PIs are demonstrated. In addition, strategies to simultaneously improve the low CTE and film toughness are discussed.

## 2. Experimental Section

### 2.1. Materials

#### 2.1.1. 5,5′-bis(2,3-norbornanedicarboxylic anhydride) (BNBDA)

**Scheme 1:**
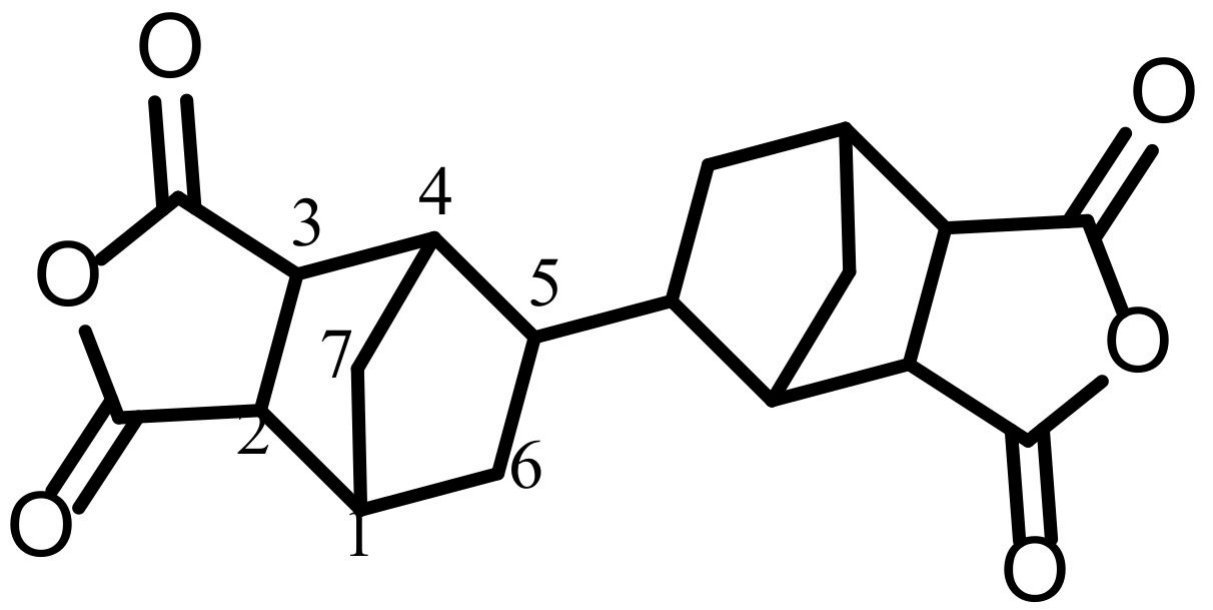
Structure of BNBDA with numbering.

BNBDA was synthesized according to the reaction schemes shown in Figure S1, as described in the patent literature [58] and purified via sublimation. The analytical data are as follows. FT-IR (KBr plate method, cm^−1^): 2985/2966/2911/2886 (C_aliph_–H stretching), 1864/1842/1775 (acid anhydride, C=O). ^1^H-NMR (400 MHz, DMSO-*d*_6_, *δ*, ppm): 3.14 (d, 2H (relative integrated intensity: 2.00H), *J* = 7.5 Hz, 3,3′-protons of the norbornane (NB) unit), 3.01 (d, 2H (2.04H), *J* = 7.5 Hz, 2,2′-protons of NB), 2.58–2.51 (m, 4H (3.82H), 1,1′,4,4′-protons of NB), 1.83–1.82 (m, 2H (1.95H), 6(H_a_),6′(H_a_)-protons (H_eq_ or H_ax_) of NB), 1.70–1.69 (m, 2H (1.91H), 6(H_b_),6′(H_b_)-protons of NB), 1.44 (d, 2H (1.93H), *J* = 11.2 Hz, 7(H_a_),7′(H_a_)-protons (away from the acid anhydride (AAn) groups) of NB), 1.19 (d, 2H (2.01H), *J* = 11.0 Hz, 5,5′-protons of NB), 0.99 (d, 2H (1.89H), *J* = 12.2 Hz, 7(H_b_),7′(H_b_)-protons (adjacent to AAn) of NB). ^13^C-NMR (100 MHz, DMSO-*d*_6_, *δ*, ppm): 175.2 (3-C=O), 174.2 (2-C=O), 49.5 (C_5_), 44.5 (C_3_), 44.0 (C_2_), 41.3 (C_4_), 41.0 (C_1_), 35.5 (C_6_), 32.4 (C_7_). Elemental analysis, Anal. Calcd. (%) for C_18_H_18_O_6_ (330.34): C, 65.45; H, 5.49. Found: C, 65.21; H, 5.27. The results correspond well to the structure of BNBDA (Scheme 1).

This product also showed a very sharp endothermic peak at 384 °C for melting by differential scanning calorimetry (DSC), as in the sublimated product (Figure S2), confirming a very high purity.

#### 2.1.2. *N*,*N*′-[2,2′-bis(trifluoromethyl)-4,4′-biphenylene]bis(4-aminobenzamide) (AB-TFMB)

**Scheme 2:**
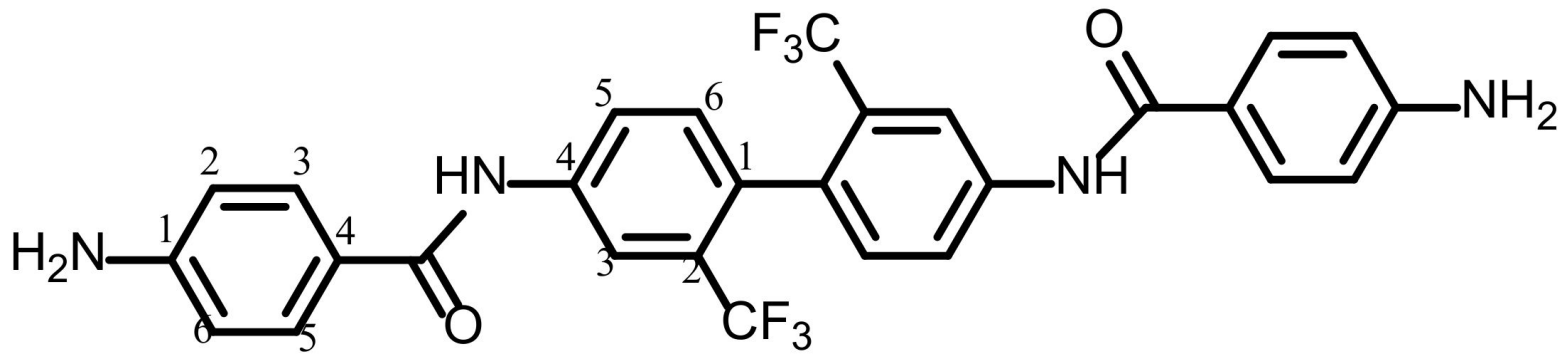
Structure of AB-TFMB with numbering.

An amide-type fluorinated diamine (AB-TFMB) was synthesized according to the procedures described in our previous studies [38,43]. The analytical data are as follows. Melting point (DSC): 317 °C. FT-IR (KBr plate method, cm^−1^): 3418 (amine, N–H stretching), 3303 (amine + amide, N–H), 3096/3039 (C_arom_–H), 1655 (amide, C=O), 1509 (1,4-phenylene), 1311 (C–F). ^1^H-NMR (400 MHz, DMSO-*d*_6_, *δ*, ppm): 10.16 (s, 2H (2.00H), NHCO), 8.33 (d, 2H (2.02H), *J* = 1.7 Hz, 3,3′-protons of the central biphenylene unit (BP)), 8.07 (dd, 2H (1.97H), *J* = 8.4, 1.6 Hz, 5,5′-protons of BP), 7.77 (d, 4H (4.05H), *J* = 8.6 Hz, 3,3′,5,5′-protons of the terminal aniline unit (AN)), 7.32 (d, 2H (2.03H), *J* = 8.4 Hz, 6,6′-protons of BP), 6.64 (d, 4H (4.02H), *J* = 8.5 Hz, 2,2′,6,6′-protons of AN), 5.86 (s, 4H (3.99H), NH_2_). Elemental analysis, Anal. Calcd. (%) for C_28_H_20_O_2_N_4_F_6_ (558.47): C, 60.22; H, 3.61; N, 10.03. Found: C, 60.00; H, 3.87; N, 9.98. The results confirm that the product is the desired diamine (AB-TFMB, Scheme 2).

#### 2.1.3. Common Monomers and Raw Materials

**Figure 4:**
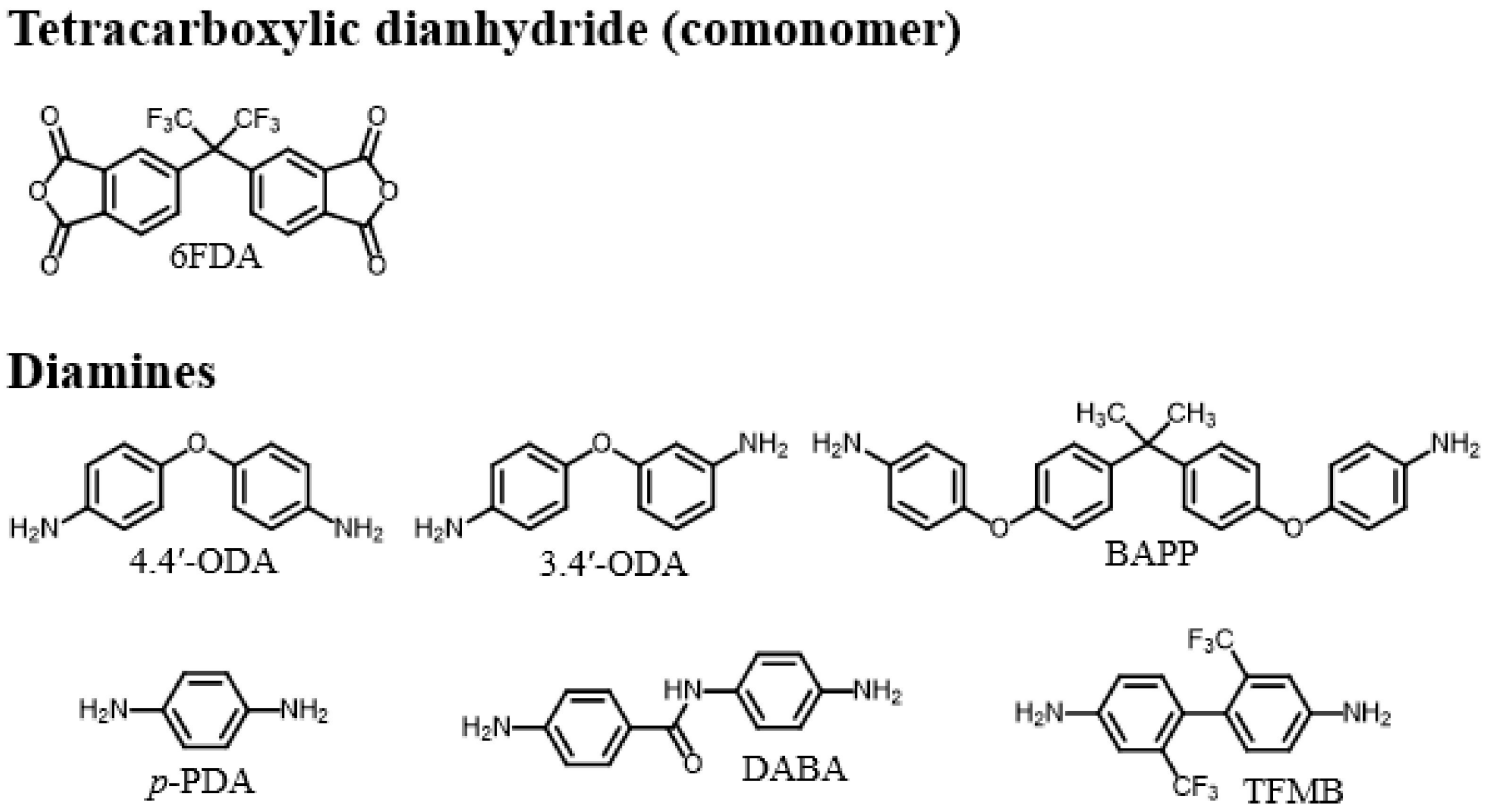
Structures and abbreviations of the common monomers used in this study.

The molecular structures of the common monomers used are shown in Figure 4. Their sources, abbreviations, and melting points, as well as those of the raw materials used, are listed in Table 1.

#### 2.1.4. BNBDA-Based Diimide Model Compounds

**Scheme 3:**
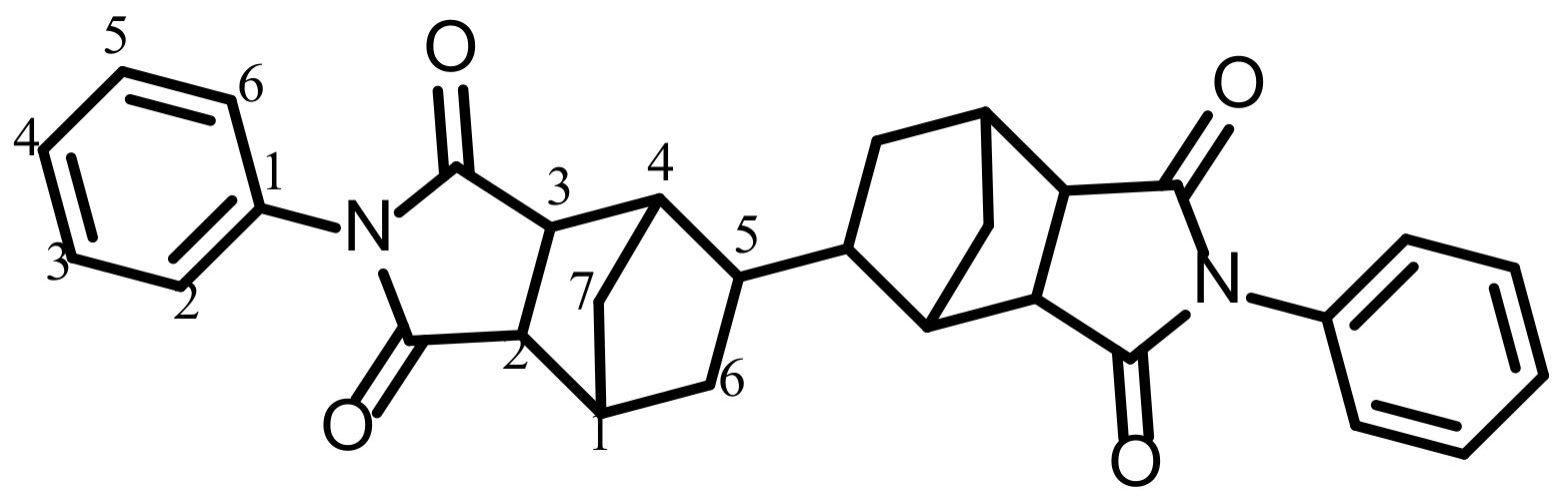
Structure of model compound (AN model) with numbering.

A low-molecular-weight BNBDA-based diimide model compound was synthesized as follows. In a three-necked 100 mL flask, BNBDA (3.0 mmol, 0.9114 g) and aniline (6.3 mmol, 0.5994 g) were dissolved in dehydrated *N*,*N*-dimethylacetamide (DMAc, 3.9 mL). The reaction mixture was stirred at room temperature in a dry nitrogen atmosphere and refluxed in an oil bath regulated at 200 °C for 4 h while gradually adding DMAc (additionally, 8 mL), then cooled to room temperature. The white crystalline precipitate formed was collected by filtration, washed with methanol, and dried at 100 °C for 12 h under vacuum (yield: 69%). Melting point (DSC): 462 °C. FT-IR (KBr plate method, cm^−1^): 3042 (C_arom_–H stretching), 2973/2930/2871 (C_aliph_–H), 1770/1708 (imide, C=O), 1493 (phenyl), 1386 (imide, *N*–Ph), 750 (imide, ring deformation). ^1^H-NMR (400 MHz, DMSO-*d*_6_, *δ*, ppm): 7.50 (t, 4H (3.93H), *J* = 7.1 Hz, 3,3′,5,5′-protons of the terminal aniline (AN) unit), 7.44 (d, 2H (1.86H), *J* = 7.3 Hz, 4,4′-protons of AN), 7.25 (d, 4H (3.91H), *J* = 7.2 Hz, 2,2′,6,6′-protons of AN), 3.04 (d, 2H (2.00H), *J* = 7.2 Hz, 3,3′-protons of NB), 2.83 (d, 2H (1.93H), *J* = 7.0 Hz, 2,2′-protons of NB), 2.58–2.50 (overlapped with a residual undeuterated DMSO proton peak at *δ* = 2.50 ppm) (m, 4H (4.64H), 1,1′,4,4′-protons of NB), 1.87 (m (br), 4H (3.94H), 6(H_a_),6′(H_a_),6(H_b_),6′(H_b_)-protons of NB), 1.40–1.37 (m, 4H (3.99H), 7(H_a_),7′(H_a_)-protons (away from the imide group) + 5,5′-protons of NB), 1.05 (d, 2H (2.00H), *J* = 9.6 Hz, 7(H_b_),7′(H_b_)-protons (adjacent to the imide groups) of NB). Elemental analysis, Anal. Calcd. (%) for C_30_H_28_O_4_N_2_ (480.56): C, 74.98; H, 5.87; N, 5.83. Found: C, 74.75; H, 6.06; N, 5.94. The results confirm that the product is the desired model compound (Scheme 3).

The product was dissolved in chloroform in a glass vessel, and it was placed into another larger sealed vessel filled by methanol to obtain a single crystal suitable for X-ray structure analysis via gradual vapor diffusion of methanol.

Other model compounds were also synthesized from BNBDA and *n*-alkylamines (*n*-butylamine and *n*-dodecylamine) in the same way as the synthetic procedures for the AN model.

#### 2.1.5. Polymerization and Film Preparation

**Figure 5:**
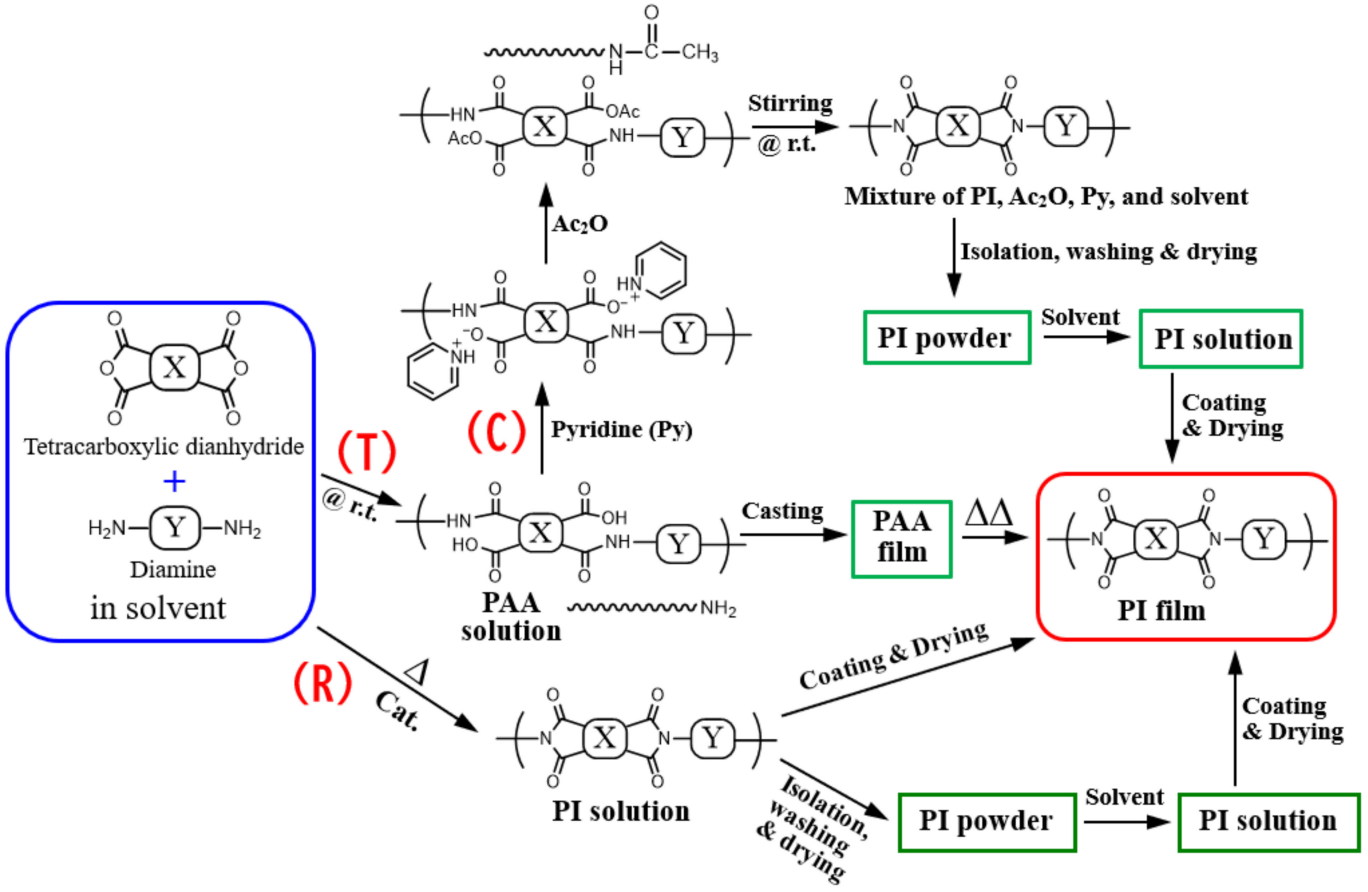
Schemes of polymerization, imidization, and film preparation via different pathways: (T) two-step process, (C) chemical imidization process, and (R) one-pot process with solution reflux.

In this study, three different routes of the polymerization and PI film preparation processes (Figure 5) were used: (1) conventional two-step process (Route T), including polyaddition, solution casting of the resultant PI precursors (poly(amic acid)s, PAAs), and thermal imidization of the PAA cast films, (2) chemical imidization process (Route C), including polyaddition of PAAs, chemical imidization in the solutions, isolation of PIs, re-dissolution of the PI powder in a fresh solvent, and the formation of PI films via solution casing, and (3) modified one-pot process (Route R), consisting of polycondensation by refluxing monomer mixtures in solutions in the presence of catalysts and coating/drying of the resultant PI solutions.

In this study, polymerization was mainly conducted by the one-pot process in a four-necked separable flask, equipped with a dry nitrogen gas inlet, and outlet connected to a silicone oil-sealed bubbler, condenser, Dean–Stark trap, and sealing mixer (sealed mechanical stirrer) (Nakamura Scientific Instruments Industry, Tokyo, Japan, UZ-SM1) with perfect sealability based on a non-contact magnetic coupling mechanism between an inner stirring rod and outer magnetic rotor. Unless otherwise stated, the one-pot polymerization was started at a total monomer content of 30 wt%, while gradually diluting as appropriate with the same solvent to ensure effective mixing. In this study, the one-pot process was modified to enhance the molecular weights of the resultant PIs as much as possible. The detailed procedures and reaction conditions are described later.

**Figure 6:**
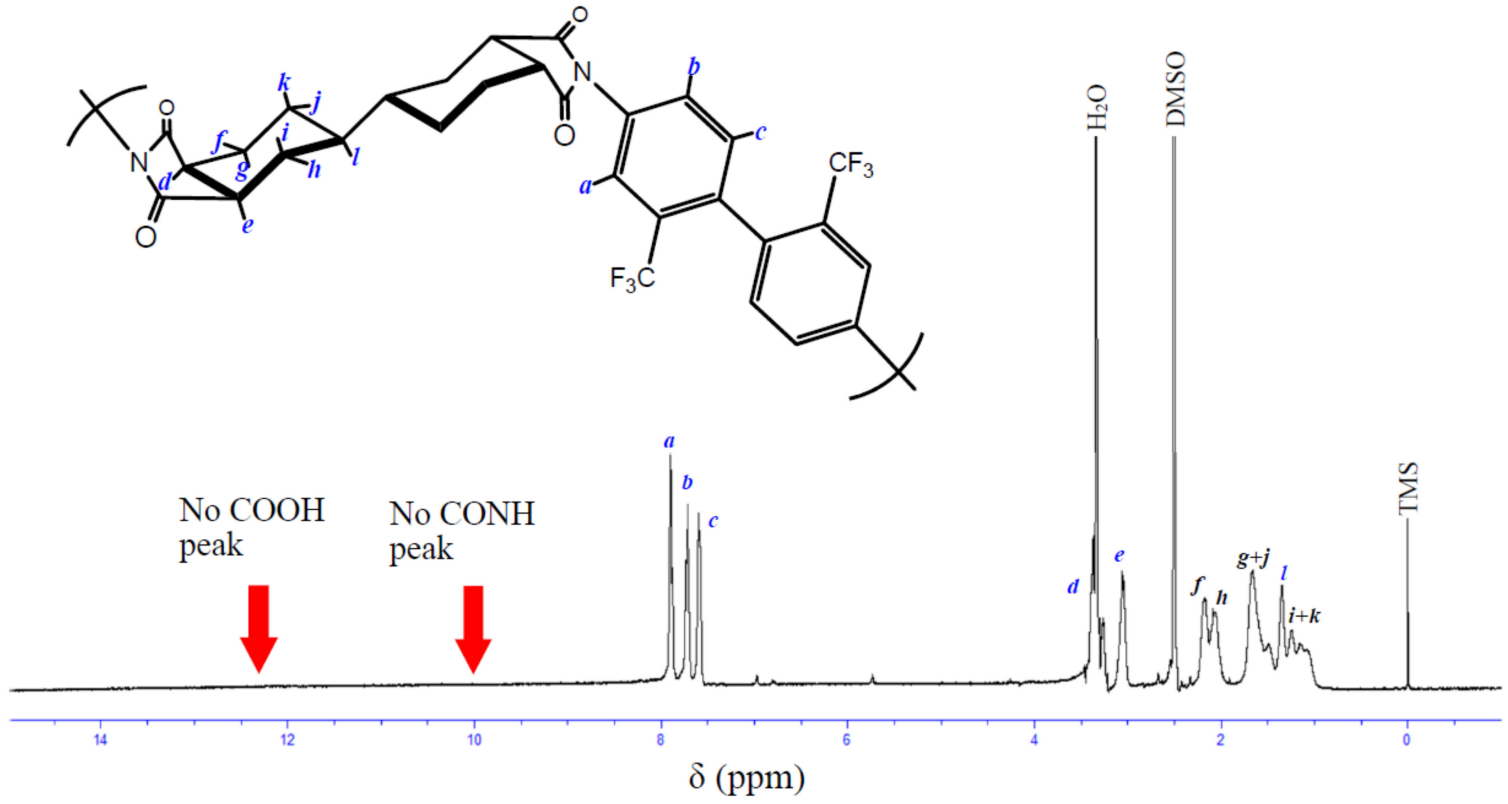
^1^H-NMR spectrum (DMSO-*d*_6_) of isolated PI powder form obtained via modified one-pot process for the H-BPDA/TFMB system.

**Figure 7:**
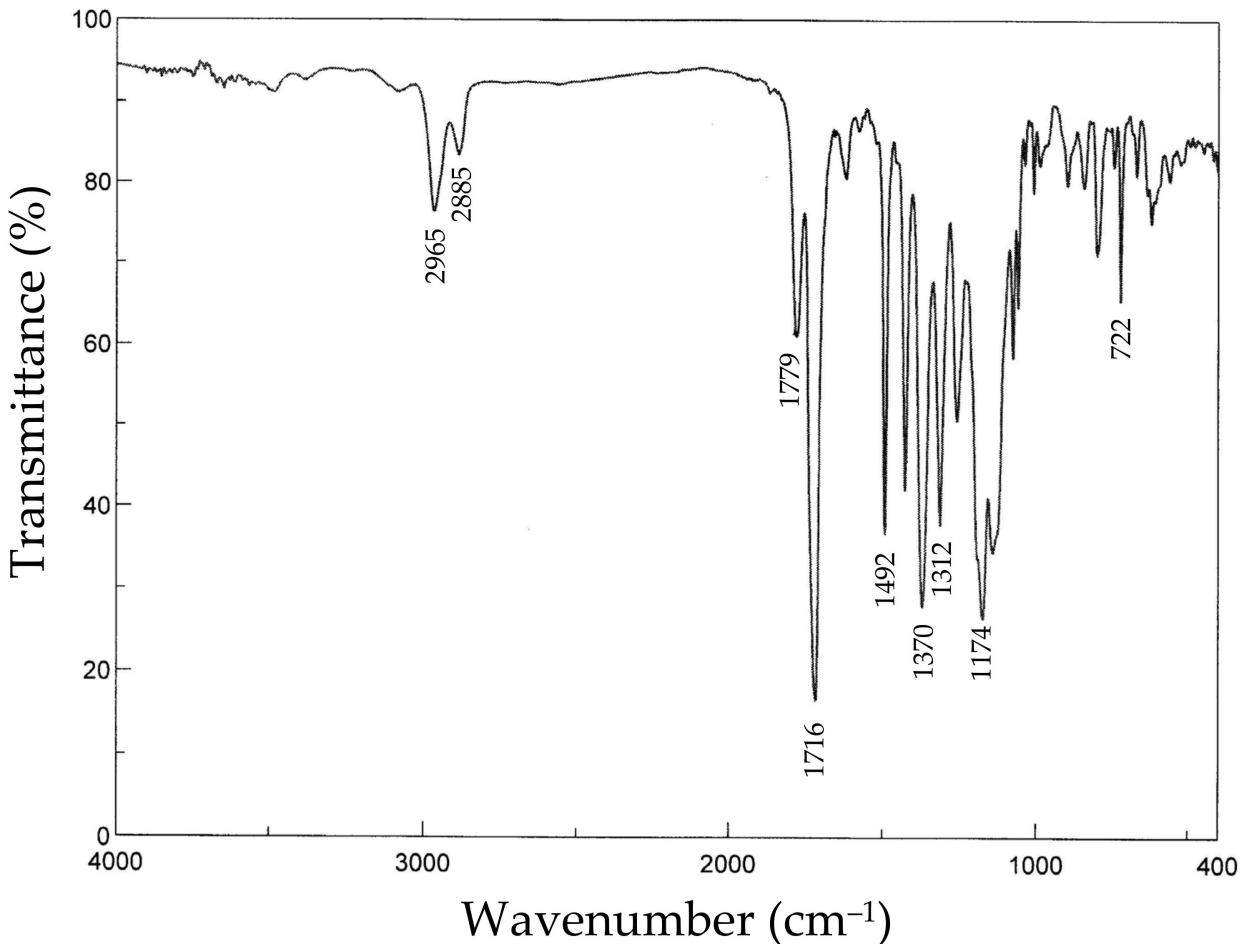
FT-IR transmission spectrum of thin cast film for BNBDA(70);6FDA(30)/TFMB copolyimide obtained via modified one-pot process.

After the one-pot polymerization, the completion of imidization was confirmed by the ^1^H-NMR spectra (DMSO-*d*_6_) from the complete disappearance of the PAA-inherent ^1^H-NMR signals; the COOH (*δ* ~12–13 ppm) and NHCO groups (*δ* ~10 ppm), as typically shown in Figure 6. The FT-IR transmission spectra of the thin films for the PIs obtained via one-pot process also confirmed complete imidization. A typical FT-IR spectrum is shown in Figure 7. The spectrum includes the specific bands (cm^−1^): 2965/2885 (C_aliph_–H), 1779/1716 (imide, C=O), 1492 (1,4-phenylene), 1370 (imide, N–C_arom_), 1312/1174 (CF_3_, C–F), and 722 (imide, ring deformation). In addition, the PAA-specific bands at ~2600 cm^−1^ (hydrogen-bonded COOH, O–H stretching) and 1680/1530 cm^−1^ (amide, C=O stretching) completely disappear.

The homogeneous PI solutions obtained via the one-pot process were used for subsequent solution casting or isolation of the PIs after adequate dilution. To remove the catalysts used in the one-pot process, the adequately diluted PI solutions were gradually poured into a large quantity of methanol or its aqueous solution, and the precipitates formed were thoroughly washed and dried. The obtained fibrous white PI powder, as shown at the bottom of Table S1, was re-dissolved in a fresh solvent for the subsequent solution casting. The resultant homogeneous PI solutions were coated on a glass substrate and dried at 80 °C for 2 h in an air-convection oven, subsequently at 150 °C for 0.5 h + 200 °C for 0.5 h + 250 °C for 1 h under vacuum on the substrate. After being peeled from the substrate, the PI films (typically 20 μm thick) were annealed at 250 °C for 1 h under vacuum to remove residual stress. In certain cases, the thermal conditions were optimized by fine-tuning to obtain a better quality of PI films.

In this work, the chemical compositions of the PAA and PI systems are represented with the abbreviations of the monomer components used (tetracarboxylic dianhydrides (A) and diamines (B)) as A/B for homopolymers and A1;A2/B1;B2 for copolymers.

### 2.2. Measurements and Characterization

#### 2.2.1. Structural Characterization

The chemical structures of BNBDA and model compounds were characterized by FT-IR (KBr plate method, Jasco, Tokyo, Japan, FT/IR 4100 infrared spectrometer), ^1^H-NMR spectra (DMSO-*d*_6_ or CDCl_3_, JEOL, Tokyo, Japan, ECP400), and elemental analysis (J-Science Lab, Kyoto, Japan, Micro Corder JM10). Their melting points were determined from the endothermic peak temperatures in the DSC thermograms using the samples put in a sealed aluminum pan on a differential scanning calorimeter (Netzsch Japan, Yokohama, Japan, DSC3100) with a heating rate of 5 °C min^−1^ in a nitrogen atmosphere. The completion of cyclodehydration (imidization) for PIs was confirmed by FT-IR and ^1^H-NMR spectroscopy.

#### 2.2.2. Inherent Viscosities and Molecular Weights

The reduced viscosities (*η*_red_) of PIs or the corresponding PAAs, which can be practically regarded as their inherent viscosities (*η*_inh_), were measured in the same solvents as those used in the polymerization at a solid content of 0.5 wt% at 30 °C on an Ostwald viscometer. 

The number (*M*_n_)- and weight (*M*_w_)-average molecular weights of highly soluble PIs in tetrahydrofuran (THF) (solid content: 0.05 wt%), pre-filtered with a PTFE-membrane filter (pore size: 0.1 μm), were determined by gel permeation chromatography (GPC) using THF as an eluent at room temperature on an HPLC system (Jasco, Tokyo, Japan, LC-2000 Plus) with a GPC column (Resonac, Tokyo, Japan, Shodex, KF-806L) at a flow rate of 1 mL min^−1^ by ultraviolet-visible detection at 300 nm (Jasco, Tokyo, Japan, UV-2075). The calibration was performed using standard polystyrenes (Shodex, SM-105).

#### 2.2.3. Linear Coefficients of Thermal Expansion (CTE)

The CTE values of PI specimens (15 mm long, 5 mm wide, and typically 20 μm thick) in the *X*–*Y* direction below the *T*_g_s were measured by thermomechanical analysis (TMA) as an average in the range of 100–200 °C at a heating rate of 5 °C min^−1^ on a thermomechanical analyzer (Netzsch Japan, Yokohama, Japan, TMA 4000) with a fixed load (0.5 g per unit film thickness in μm, i.e., 10 g load for 20 μm thick films) in a dry nitrogen atmosphere. In this case, after the preliminary first heating run up to 120 °C and successive cooling to room temperature in the TMA chamber, the data were collected from the second heating run to remove the influence of adsorbed water.

#### 2.2.4. Heat Resistance

The glass transition temperatures (*T*_g_s) of PI films were determined from the peak temperature of the loss energy (*E*″) curves by dynamic mechanical analysis (DMA) at a heating rate of 5 °C min^−1^ on the TMA instrument (as before). The measurements were conducted at a sinusoidal load frequency of 0.1 Hz with an amplitude of 15 gf in a nitrogen atmosphere.

The thermal and thermo-oxidative stability of PI films were evaluated from the 5% weight loss temperatures (*T*_d_^5^) by thermogravimetric analysis (TGA) on a thermo-balance (Netzsch Japan, Yokohama, Japan, TG-DTA2000). TGA was performed at a heating rate of 10 °C min^−1^ in a dry nitrogen and/or air atmosphere. The small weight loss due to the desorbed water around 100 °C during the TGA heating runs was compensated by offsetting at 150 °C to 0% weight loss for the data analysis.

#### 2.2.5. Optical Transparency

The light transmission spectra of the PI films (typically 20 μm thick) were measured on an ultraviolet-visible spectrophotometer (Jasco, Tokyo, Japan, V-530) in the wavelength (*λ*) range of 200–800 nm. The light transmittance at 400 nm (*T*_400_) and the cut-off wavelength (*λ*_cut_) at which the transmittance becomes substantially zero were determined from the spectra. 

The yellowness indices (YI, ASTM E 313) for PI films were determined from the spectra under a standard illuminant of D65 and a standard observer function of 2° using a color calculation software (Jasco, Tokyo, Japan) on the basis of the relationship:

YI = 100 (1.2985*x* − 1.1335*z*)/*y* (1)

where *x*, *y*, and *z* are the CIE tristimulus values. YI takes zero for an ideal white/transparent sample. 

The total light transmittance (*T*_tot_, JIS K 7361-1) and the diffuse transmittance (*T*_diff_, JIS K 7136) of PI films were measured on a double-beam haze meter equipped with an integrating sphere (Nippon Denshoku Industries, Tokyo, Japan, NDH 4000). The haze (turbidity) of PI films was calculated from the relationship:

Haze = (*T*_diff_/*T*_tot_) × 100 (2)

#### 2.2.6. Birefringence

The in-plane (*n*_in_ or *n*_xy_) and out-of-plane (*n*_out_ or *n*_z_) refractive indices of PI films were measured with a sodium lamp at 589.3 nm (*D*-line) on an Abbe refractometer (Atago, 4T, *n*_D_ range: 1.47–1.87) equipped with a polarizer using a contact liquid (sulfur-saturated methylene iodide, *n*_D_ = 1.78–1.80) and a test piece (*n*_D_ = 1.92). The birefringence of PI films, which represents the relative extent of chain alignment in the *X*–*Y* direction, was calculated from the relationship:

Δ*n*_th_ = *n*_in_ − *n*_out_ (3)

#### 2.2.7. Mechanical Properties

The tensile modulus (*E*), tensile strength (*σ*_b_), and elongation at break (*ε*_b_) of PI specimens (film dimension: 30 mm long, 3 mm wide, typically 20 μm thick; specimen numbers > 15) were measured on a mechanical testing machine (A & D, Tokyo, Japan, Tensilon UTM-II) at a cross-head speed of 8 mm min^−1^ at room temperature. The specimens were cut from high-quality film samples free of any defects, such as fine bubbles. The data analysis was carried out using a data processing program (Softbrain, UtpsAcS Ver. 4.09, Tokyo, Japan).

#### 2.2.8. Water Uptake

The degrees of water absorption (*W*_A_, %) of the PI films were determined according to the JIS K 7209 standard using Equation (4):

*W*_A_ = [(*W* − *W*_0_)/*W*_0_] × 100 (4)

where *W*_0_ is the weight of a film sample (>0.1 g) just after vacuum-drying at 50 °C for 24 h, and *W* is the weight of the film immersed in water at 23 °C for 24 h and carefully blotted dry with tissue paper. 

#### 2.2.9. Solubility

The solubility of PIs was qualitatively examined using their powder samples (the bottom of Table S1) prepared via the modified one-pot process by observing whether the samples (10 mg) were completely dissolved in various solvents (1 mL) in a test tube without heating (first step). For the insoluble samples at room temperature, they were heated at a temperature established for each solvent (2nd step), and the presence/absence of dissolution was observed.

#### 2.2.10. Single-Crystal X-ray Structural Analysis

X-ray structural analysis for the BNBDA-based model compounds was conducted at 100 K on a single-crystal X-ray diffractometer (Bruker Japan, Yokohama, Japan, APEXII) with a CCD area detector using a graphite-monochromated Mo*K*α radiation source (*λ* = 0.71073 Å). The three-dimensional intensity data collected were analyzed using SHELXS97 and refined using SHELXL97.

#### 2.2.11. Liquid Crystallinity

Liquid crystallinity of the model compounds was observed on an Olympus BX51 (Tokyo, Japan) polarizing optical microscope (POM) equipped with a digital camera (Nikon Coolpix 950) and a temperature-controllable hot stage (Mettler Toledo, Columbus, OH, USA, FP82HT hot stage and FP 90 central processor).

## 3. Results and Discussion

### 3.1. Importance of Thermal Dimensional Stability and Difficulty in Simultaneously Achieving Low CTE, High Optical Transparency, Ductility, and Solution Processability

**Figure 8:**
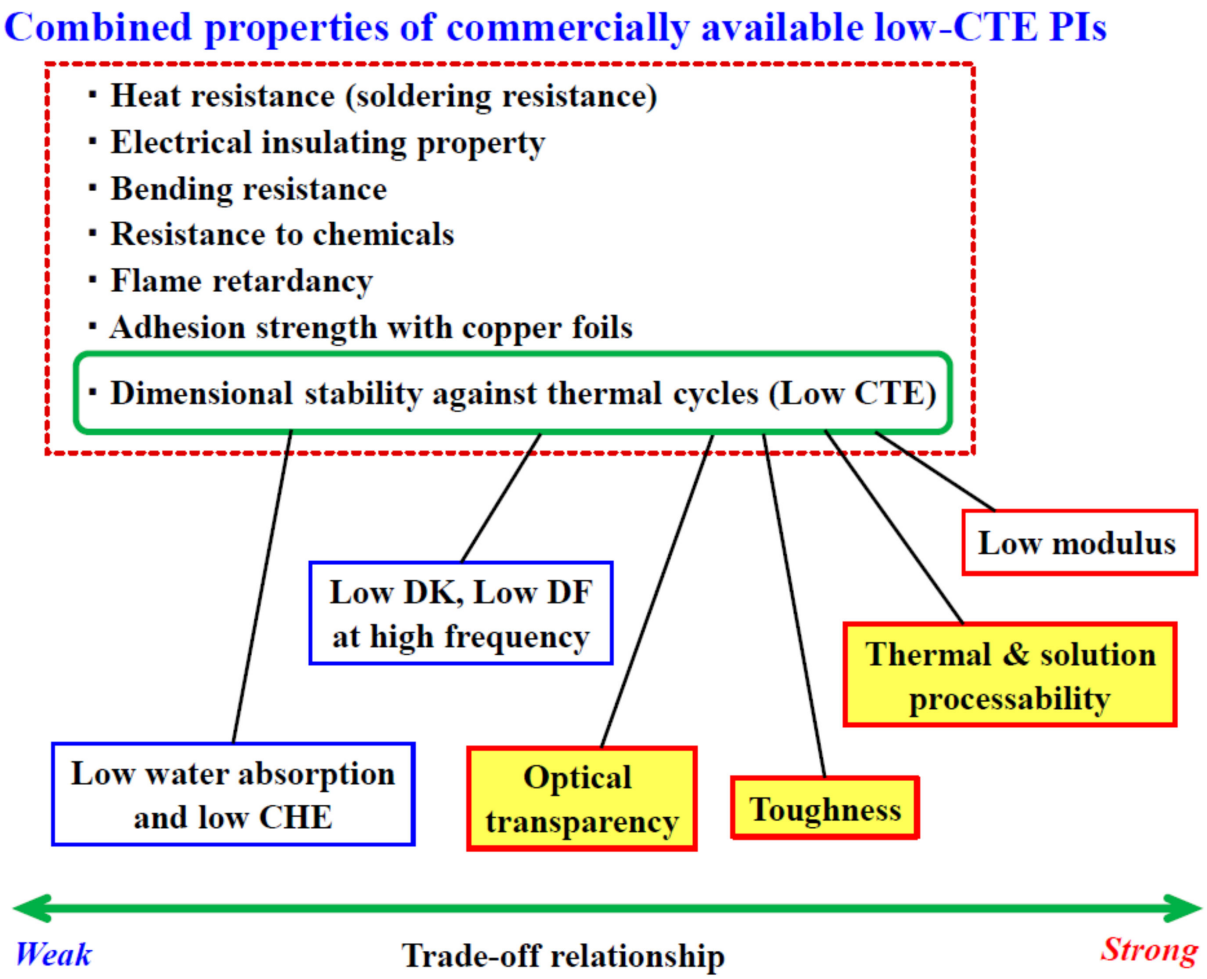
The combined properties of commercially available low-CTE polyimide films and the difficulty in simultaneously achieving low CTE and other desired properties, relating to the trade-off in principle between them. The yellow-highlighted items represent the challenging goal established in this study.

To apply colorless PIs as flexible plastic substrate alternatives to conventional inorganic glass substrates in various optoelectronic devices, they are required to have not only a high *T*_g_ but also high dimensional stability during multiple heating–cooling cycles in the device manufacturing processes, specifically, a low CTE in the *X*–*Y* direction in the glassy temperature regions (*T* < *T*_g_). Otherwise, the significant repeated thermal expansion–contraction (with hysteresis in some cases) in the plastic substrates during the thermal cycles causes serious problems, including misalignment and adhesion failure of various micro-components, laminate warpage, and transparent electrode breakdown. In recent years, combinations of a low CTE and other important properties, which are often in a trade-off relationship, have become necessary in some cases, as shown in Figure 8. In this figure, the properties located on the right are more difficult to achieve in principle, together with a low CTE. 

Previously, we attempted to overcome the trade-off between a low CTE and high thermoplasticity [59] and that between a low CTE and low tensile modulus [60]. In the present study, we established targets to simultaneously achieve the yellow-highlighted properties (high optical transparency, suitable film toughness, and excellent solution processability) with low CTE properties.

In principle, simultaneously achieving low CTE and high optical transparency is not always the trade-off. However, in fact, this issue is not easy to solve because the use of cycloaliphatic monomers to ensure film transparency often increases CTE, suggesting the importance of their steric structures in reducing the CTE of the resultant PI films [30,31,32,38,45,47]. On the other hand, there is the trade-off between low CTE and excellent solution processability because the molecular design for ensuring a low CTE (i.e., to enhance main-chain linearity/rigidity) often results in a significant decrease in solubility, and *vice versa* [61,62]. There is similar difficulty in simultaneously achieving low CTE and high film toughness. This is because linear/rigid main-chain structures favorable for a low CTE usually contribute to a decrease in chain entanglement, which often deteriorates film toughness, as typically shown in a rod-like PI system derived from PMDA and *p*-phenylenediamine (*p*-PDA) [63]. In contrast, the PMDA/4,4′-ODA PI film (4,4′-ODA = 4,4′-oxydianiline), which includes rotatable ether linkages, exhibits extremely high toughness but does not show a low CTE [54].

In the present study, we propose strategies for simultaneously achieving a low CTE, high optical transparency, suitable toughness, and excellent solution processability.

### 3.2. Importance of Excellent Solubility

Highly soluble PI systems are compatible with simple film preparation processes using homogeneous PI solutions via chemical imidization (C) or one-pot polycondensation (R) in addition to the conventional two-step process (Figure 5). We previously reported that PI films (C) tended to show higher optical transparency and lower CTEs than their counterparts (T) with the same chemical composition and proposed their mechanisms [62]. This suggests that excellent solubility is the key to the success or failure of the present mission. However, there is a limitation to the application of the chemical imidization process: the opportunity to enhance the molecular weights of the obtained PIs is only available during polyaddition (PAA formation) before chemical imidization because the molecular weights do not usually increase during chemical imidization [20]. Therefore, systems without sufficiently high polyaddition reactivity, even if they have excellent solubility, are not compatible with the film preparation process via chemical imidization because of the predicted insufficient film ductility. However, if high solubility is ensured, the one-pot process in the presence of catalysts can solve this problem because of its striking effect on molecular weight enhancement. As discussed later, the combination of the copolymerization approach for dramatically improving the solubility and the application of a condition-adjusted one-pot process for maximizing the molecular weight was indispensable for achieving the goal established in this study.

### 3.3. Steric Structure of BNBDA

**Figure 9:**
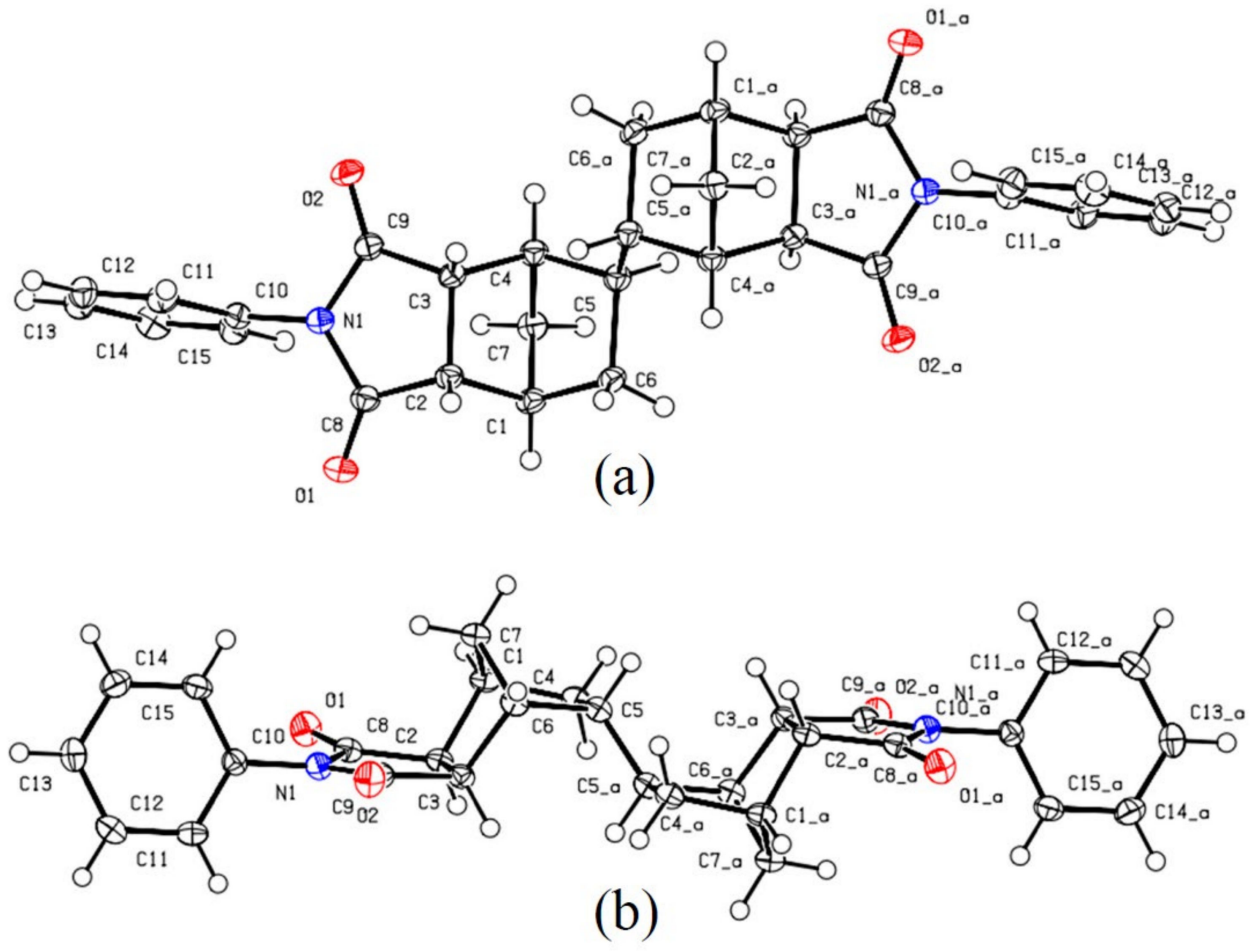
Steric structure determined by single-crystal X-ray analysis for the diimide compound obtained from BNBDA and aniline: (**a**) top view and (**b**) side view of NB rings.

Attempts to obtain a single crystal of BNBDA were unsuccessful. Instead, a low-molecular-weight diimide compound (AN-model) was synthesized and crystallized. The steric structure and crystallographic data obtained by the single-crystal X-ray structural analysis are shown in Figure 9 and Table 2, Table 3, Table 4, Table 5 and Table 6. As is evident from the side view in Figure 9b, the BNBDA-based diimide (BNBDI) unit has a crank-shaft-like and highly linear steric structure. This suggests that the BNBDI units incorporated into the PI main chains have a similar steric structure. An AN-model analog, C_4_-model, synthesized from BNBDA and *n*-butylamine in a similar manner, was found to have a very similar steric structure in the BNBDI unit by X-ray analysis.

In addition, the liquid crystallinity of the model compounds (AN-model and C_4_-model) was investigated using POM. However, they did not show optically anisotropic thermotropic liquid–crystalline (LC) textures above their melting points in both the heating and cooling processes. Considering that sufficient fluidity above the melting points is indispensable for the formation of LC phases, the non-liquid crystallinity observed here is probably attributed to their very high melting points (462 °C for the AN-model and 214 °C for C_4_-model at a heating rate of 5 °C min^−1^ on DSC); when the melting points were too high, the LC phase became unstable, owing to a thermal randomizing effect increased at high temperatures. In addition, the LC phase can appear only when there is a suitable balance in one molecule between the structural rigidity of the mesogenic units and the flexibility of the rotatable soft units.

On the other hand, the extension of the flexible alkyl chain from *n*-butyl (C_4_) to *n*-dodecyl (C_12_) groups significantly reduced the melting point (142 °C for C_12_-model, Figure S3a). Under the POM observation, the onset of flowing at 134.0 °C, a thermotropic LC phase in the range of 137–139 °C, and a clearing point at 139.6 °C were observed in the heating process. Furthermore, in the cooling process from an isotropic state at 160 °C, an LC phase with fluidity was observed in the range of 136–133 °C, as shown in Figure S3b. The results probably reflect that the BNBDI unit in the C_12_-model behaved as a mesogenic unit, which generally consists of a rigid and extended structure. This interpretation also suggests that the BNBDI unit prefers to adopt a well-extended structure (Figure S3c(i)) rather than a distorted structure arising from a conformational change around the NB–NB bond (Figure S3c(ii)). Accordingly, when BNBDA was combined with adequate aromatic diamines with rigid and linear structures to ensure overall chain linearity, and sufficiently high molecular weights of PIs were obtained, it is likely that a low CTE is obtained while maintaining high optical transparency, although the concomitant deteriorations of the solution processability and film toughness are concerned.

### 3.4. Speculated Steric Structure of H-BPDA

In this study, a hydrogenated product of 3,3′,4,4′-biphenyltetracarboxylic dianhydride (*s*-BPDA), i.e., H-BPDA, was used as a comparative system. The crystallization of H-BPDA-based model compounds was unsuccessful. Hence, the steric structure of H-BPDA used in this study was predicted by comparing its spectral data that we measured with those reported in the literature [33,42,64]. 

Shiotani et al. [33,64] proved that a single crystal obtained via catalytic hydrogenation of 3,3′,4,4′-biphenyltetracaboxylic acid tetramethyl ester, hydrolysis of the reductant, cyclodehydration, and crystallization has a highly distorted steric structure, i.e., *rel*-(1R,1′*S*,3R,3′*S*,4S,4′*R*)-dicyclohexyl-3,3′,4,4′-tetracarboxylic dianhydride (*cis*-DCDA, Figure S4a). On the other hand, a stereoisomer of *cis*-DCDA, *rel*-(1R,1′*S*,3R,3′*S*,4R,4′*S*)-dicyclohexyl-3,3′,4,4′-tetracarboxylic dianhydride (*trans*-DCDA, Figure S4b), which has a steric structure with higher planarity and linearity than *cis*-DCDA, can be obtained by intentionally isomerizing the reductant of the tetramethyl ester. The *cis*-DCDA showed a reasonable ^1^H-NMR spectrum where the signal of 3,3′-axial protons on the cyclohexane (CH) units were clearly separated from that of the 4,4′-equatorial protons, reflecting their different magnetic environments [64]. H-BPDA used in the present study also exhibited a very similar ^1^H-NMR spectrum with the above-mentioned feature consisting of the 4,4′-equatorial proton signal peak centered at 3.50 ppm (not well resolved) and the 3,3′-axial proton signal centered at 3.13 ppm (not well resolved)], as shown in Figure S5. A similar separated proton signal pattern was also observed in the *cis*-DCDA/4,4′-ODA polyimide, in contrast, *trans*-DCDA/4,4′-ODA polyimide showed a spectral overlap between the 3,3′- and 4,4′-proton signals, reflecting their almost identical magnetic environments (axial protons for all) [33,64]. Our PI counterpart (H-BPDA/4,4′-ODA) also exhibited a clearly separated proton signal pattern (Figure 6), similar to that of the former (*cis*-DCDA/4,4′-ODA). These results suggest that H-BPDA used in this study has essentially the same steric structure as *cis*-DCDA. In addition, as mentioned later, the H-BPDA/4,4′-ODA PI film exhibited a *T*_g_ close to that of the *cis*-DCDA/4,4′-ODA film reported in the literature [33].

### 3.5. Polyaddition Reactivity of BNBDA with Aromatic Diamines

First, the polyaddition reactivity of BNBDA with various aromatic diamines was investigated. Table 7 summarizes the polyaddition conditions, the success/failure of the reactions, and the reduced viscosities of the resulting PAAs. DMAc is empirically known to be superior to *N*-methyl-2-pyrrolidone (NMP) in suppressing the coloration of the resultant PI films [31,38]. This is probably due to the higher volatility of the former (shorter solvent-staying time in the films during thermal imidization); more volatile solvents are advantageous for suppressing a residual unknown colored product from the partially decomposed solvents. Therefore, DMAc was used in the first attempt at polyaddition.

Even BNBDA without additional purification by sublimation showed relatively good reactivity with 4,4′-ODA (#0) and led to a homogeneous and viscous solution of PAA with *η*_red_ = 0.79 dL g^−1^, although it was slightly lower than an empirical rough criterion for sufficiently high molecular weights (*η*_red_ = 1 dL g^−1^). On the other hand, the use of sublimated BNBDA discernibly enhanced its *η*_red_ value (up to 1.05 dL g^−1^, #1), although there was no substantial difference in the chemical purity of BNBDA before (m.p. 384 °C by DSC) and after sublimation (m.p. 385 °C, Figure S2), as suggested by comparisons of their ^1^H-NMR spectra and elemental analysis data. A possible reason for the slightly increased *η*_red_ value is that a trace amount of the hydrolyzed portion present in BNBDA was likely cyclodehydrated completely during sublimation. Therefore, sublimated BNBDA was consistently used in this study.

In contrast to BNBDA, a BNBDA analog, H-BPDA, showed very high polyaddition reactivity with 4,4′-ODA (#8) and led to a homogeneous and highly viscous solution of PAA with a much higher *η*_red_ (2.99 dL g^−1^). The reason for the lower polyaddition reactivity of BNBDA than that of H-BPDA is not clear.

Contrary to our initial expectations, the equimolar reaction mixture of BNBDA and 2,2-bis[4-(4-aminophenoxy)phenyl]propane (BAPP) (#2) in DMAc remained inhomogeneous even after prolonged stirring, mild heating, and dilution. This is a very rare case because polyaddition usually proceeds smoothly regardless of the TCDAs when flexible ether-containing BAPP is used. This failure may be attributed to the poor solubility of the oligo amic acid (oligo-AA) formed in DMAc. Fortunately, by replacing DMAc with NMP, a homogeneous PAA solution was finally obtained, although the resultant *η*_red_ value (*η*_red_ = 0.69 dL g^−1^) was not as high as expected.

To obtain low-CTE PI films, a typical rigid diamine, *p*-PDA, was combined with BNBDA (#3). The reaction mixture was homogenized once; however, gelation occurred in both DMAc (#3) and NMP (#3′), and soft heating and dilution were ineffective for homogenization. This hindered the subsequent film preparation (solution casting) process. A similar gelation phenomenon was previously observed during the polyaddition of *p*-PDA and an H-PMDA isomer, 1*S*,2*S*,4*R*,5*R*-cyclohexanetetracarboxylic dianhydride (H′-PMDA), in DMAc [32]. The gelation observed in BNBDA/*p*-PDA results from the formation of poorly soluble oligo-AA. Another typical rigid diamine, 4,4′-diaminobenzanilide (DABA), was used (#4). However, the reaction caused similar gelation in both DMAc (#4) and NMP (#4′), and subsequent film preparation was disturbed.

TFMB (#5), a limited diamine advantageous for achieving high optical transparency, low CTE, and high solubility, was selected in combination with BNBDA. The reaction mixture remained inhomogeneous up to a reaction period of 7 days; however, after that, the precipitates started to dissolve and finally became homogeneous after stirring for an additional 2 days (total 9 days) at room temperature. The *η*_red_ value of the resultant PAA was too low (0.39 dL g^−1^) to form a ductile, free-standing film; the PI film prepared via the two-step process was so brittle that it was difficult to evaluate its mechanical properties. Chemical imidization using this PAA solution resulted in PI with a further decreased *η*_red_ value (0.13 dL g^−1^), which expectedly provided a very brittle cast film including cracks. Thus, neither the conventional two-step process nor the chemical imidization process was successful in obtaining a ductile, free-standing BNBDA/TFMB PI film.

**Figure 10:**
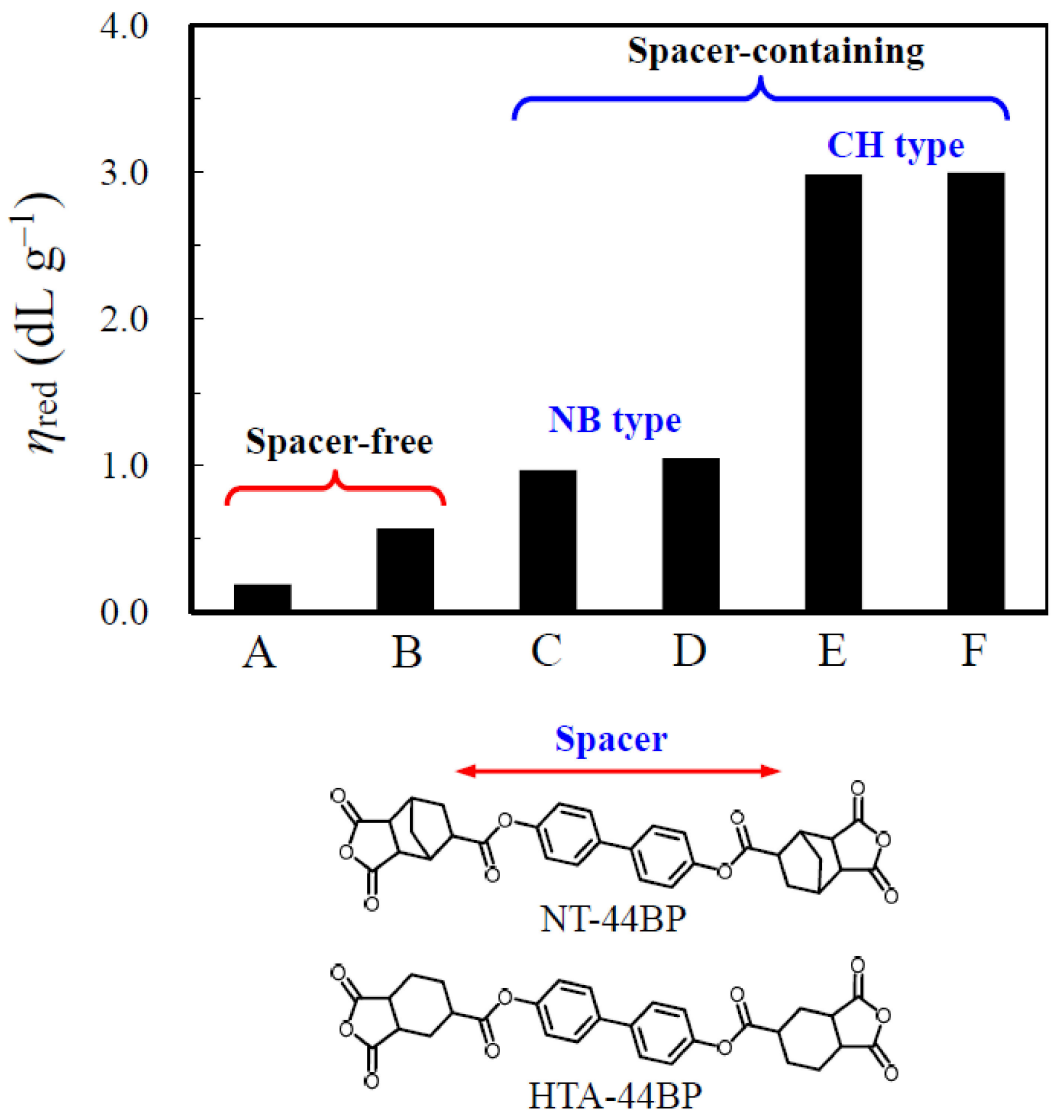
The *η*_red_ (PAA)-based relative polymerizability of cycloaliphatic tetracarboxylic dianhydrides (A: BTA, B: H-PMDA, C: NT-44BP, D: BNBDA, E: H-BPDA, and F: HTA-44BP) with 4,4′-ODA. Polyaddition was conducted in DMAc or NMP at room temperature at an initial monomer content of 30 wt%, and the reaction mixture was gradually diluted with a minimal quantity of the same solvent to ensure effective magnetic stirring.

Figure 10 shows a comparison of *η*_red_-based polyaddition reactivity of BNBDA and other related cycloaliphatic TCDAs with 4,4′-ODA. For BTA and H-PMDA, in which two functional groups are connected to the same cycloaliphatic unit (bridged and non-bridged CH units), it was difficult to obtain a sufficiently high *η*_red_ value (≥~1 dL g^−1^). We previously proposed a hypothesis for the relatively low polyaddition reactivity of H-PMDA, which is related to steric hindrance arising from the adjacent functional groups with an all-*exo*-configuration [30]. Indeed, bis-cycloaliphatic-type (BNBDA and H-BPDA) and spacer-containing TCDAs (NT-44BP [65], HTA-44BP [29]), where the proximity of the two functional groups is reduced, displayed distinctly enhanced *η*_red_ values. Figure 10 also suggests a structural effect of the cycloaliphatic units connected to the functional groups (that is, cyclohexane (CH) or norbornane (NB)); an NB-type TCDA, BNBDA, leads to a distinctly lower *η*_red_ value than its CH-type counterpart (H-BPDA). A similar situation was observed when comparing NT-44BP with HTA-44BP, although the mechanism is not clear. A possible explanation for this is that the bridging group of the NB unit can act as a sterically hindering group when the amine-terminated oligomer approaches an adjacent functional group.

Thus, BNBDA was found to have significant disadvantages during polyaddition: lower solubility of the oligo-AAs formed and lower reactivity, peculiar to NB-type TCDAs.

### 3.6. Properties of BNBDA-Based PI Films Prepared via Two-Step Process

#### 3.6.1. Features of BNBDA/4,4′-ODA System

Table 8 summarizes the properties of the homo BNBDA-based PI films (T). The BNBDA/4,4′-ODA film (#0T) obtained from non-sublimated BNBDA and 4,4′-ODA under thermal condition A (PAA solution coating + half-drying at 80 °C for 2 h + imidization at 150, 200, 250, and 300 °C for each 0.5 h on the substrate under vacuum + annealing at 300 °C for 1 h without the substrate under vacuum) showed high optical transparency. However, the PI films were too brittle for mechanical testing. In addition, the PI powder sample isolated via chemical imidization temporarily provided a homogeneous solution by re-dissolution upon heating in cyclopentanone; however, the solution underwent gelation during standing at room temperature.

On the other hand, the use of sublimated BNBDA somewhat increased the *η*_red_ value (up to 1.05 dL g^−1^) of the resultant PAA. However, the film ductility was poorly improved when the PI film was prepared under thermal condition A. Then, the thermal conditions were optimized as follows (condition B: PAA solution coating + half-drying at 60 °C for 2 h + imidization at 200, 250, 300, and 330 °C for each 0.5 h on the substrate under vacuum + annealing at 300 °C for 1 h without the substrate under vacuum). Consequently, a ductile PI film (*ε*_b max_ = 18.5%) was obtained. This PI film (#1T) also exhibited very high optical transparency (YI = 1.4, haze = 0.63%); however, it did not show low-CTE properties, inevitably owing to the use of the flexible ether-containing diamine (4,4′-ODA). The following remarkable feature was observed: despite the use of 4,4′-ODA, the resultant PI film unexpectedly exhibited an extremely high *T*_g_ (361 °C). This probably reflects the local structural rigidity of the BNBDI units in the main chains.

**Figure 11:**
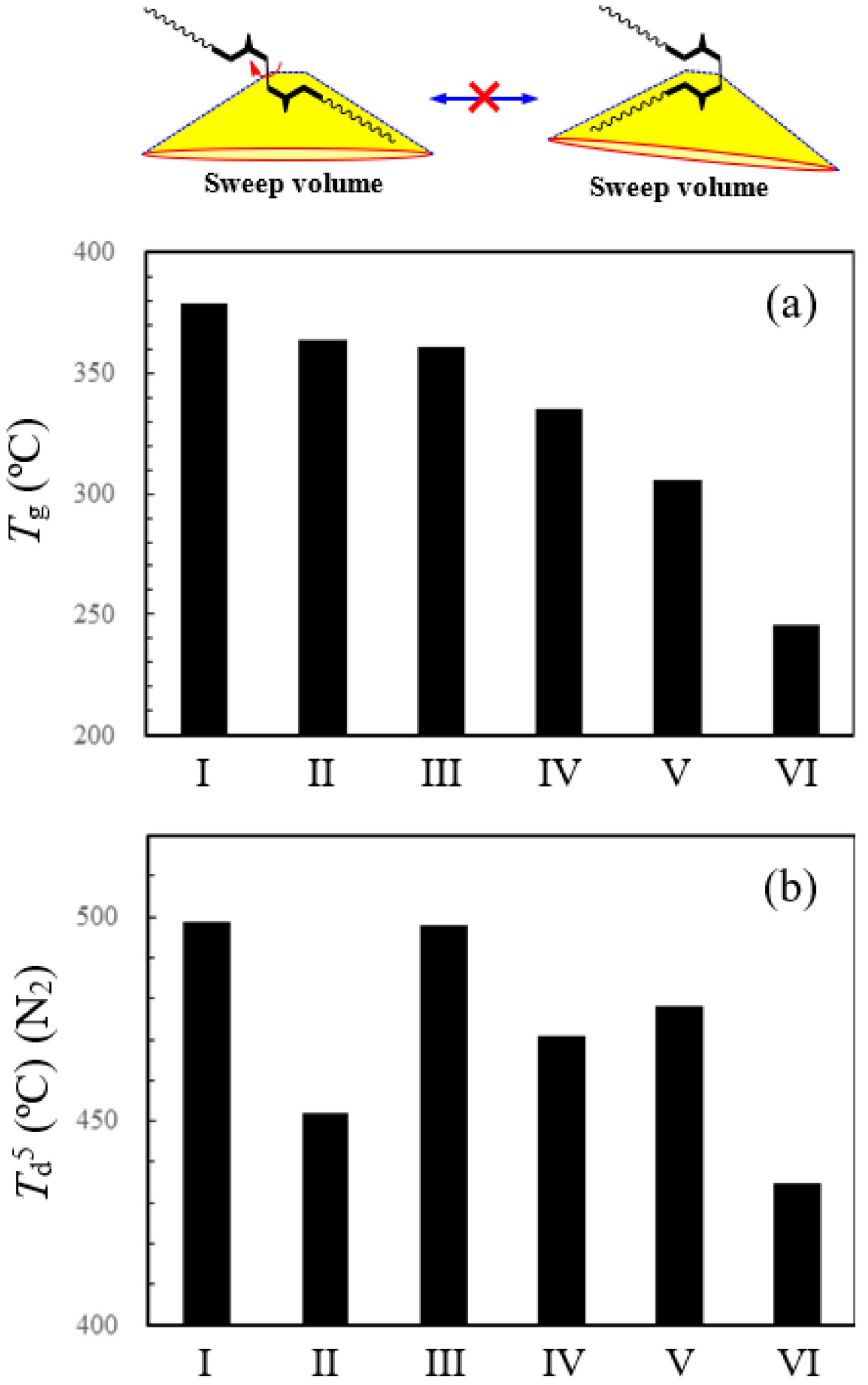
The *T*_d_^5^ in N_2_ (**a**) and *T*_g_ (by DMA) (**b**) of the PI films (T) obtained from 4,4′-ODA and various cycloaliphatic tetracarboxylic dianhydrides: (I) H-BTA, (II) CBDA, (III) BNBDA, (IV) H-PMDA, (V) OHADA, and (VI) H-BPDA. The figure at the top represents a large sweep volume required for internal rotation around the NB–NB bond in BNBDI units.

The *T*_g_ value of the BNBDA/4,4′-ODA film (#1T) was compared to those of its counterparts derived from other related cycloaliphatic TCDAs, as shown in Figure 11a. The *T*_g_ decreased in the following order depending on the TCDA used: H-BTA > CBDA > BNBDA > H-PMDA > OHADA >> H-BPDA. The PIs based on H-BTA [56] and CBDA [43] had the highest *T*_g_ values. The latter is closely related to the absence of internal rotationability in the four-membered cyclobutane portion of the CBDA-based diimide (CBDI) units. On the other hand, the former very likely results from the significant inhibition of internal rotation in the CH portion by the bridging group. The higher contents of imide C=O groups, as the origin of dipole–dipole attractive interactions [66,67], also contribute to an increase in *T*_g_, as in systems using lower-molecular-weight monomers (typically CBDA). However, even though BNBDA is a bis-cycloaliphatic-type TCDA with a rotatable NB–NB single bond, the BNBDA/4,4′-ODA PI film exhibited an extremely high *T*_g_ (361 °C), comparable to that of the CBDA/4,4′-ODA film (*T*_g_ = 364 °C [43]). Surprisingly, the former was 90–100 °C higher than that of the corresponding aromatic analog (*s*-BPDA/4,4′-ODA, *T*_g_ = 260–270 °C [68]). This suggests that the internal rotation around the NB–NB bond in the BNBDI unit is much more suppressed than that around the Ph–Ph bond in the *s*-BPDI unit. The lower rotational flexibility of the former is probably closely related to the fact that the NB–NB (C_5_–C_5′_) single bond is in the axial direction of each other, as shown in Figure 9b; thereby, the internal rotation around the NB–NB bond involves a large translational motion together with the connecting chains (i.e., large sweep volume) while shoving the surrounding molecules, as depicted at the top of Figure 11a. Therefore, the rotation around the C_5_–C_5′_ single bond is assumed to be significantly inhibited, unlike its *s*-BPDA-based counterpart, which has a much smaller sweep volume.

On the other hand, the use of OHADA resulted in a distinctly decreased *T*_g_ in relation to that of the above-mentioned ultrahigh-*T*_g_ PI groups (H-BTA-, CBDA-, and BNBDA-based systems), as shown in Figure 11a, probably owing to disturbed close chain stacking, consequently weakened dipole–dipole interactions arising from a highly distorted, and non-planar steric structure of the OHADA-based diimide (OHADI) units [47].

A BNBDA/4,4′-ODA analog, the H-BPDA/4,4′-ODA system (#8T) showed the lowest *T*_g_ (246 °C) among the systems compared in Figure 11a, probably reflecting the combined effect of the allowed conformational changes, including the chair–boat interconversion, in the H-BPDA-based diimide (H-BPDI) units at elevated temperatures and the highly distorted steric structure of H-BPDI units (Figure S4a). This *T*_g_ value was comparable to that of the counterpart derived from a typically flexible TCDA, 4,4′-oxydiphthalic anhydride (ODPA) (*T*_g_ = 250 °C [69])

The measured *T*_g_ for the H-BPDA/4,4′-ODA (246 °C by DMA at 0.1 Hz, Table 8) was also approximate to that for the *cis*-DCDA/4,4′-ODA film reported in the literature (259 °C by DSC [33] and 256 °C by DMA at 1 Hz [42]). These results do not conflict with the aforementioned prediction, based on the ^1^H-NMR spectral similarity, that H-BPDA used in this study has essentially the same steric structure as *cis*-DCDA [33,64].

Thus, the novel bridge-containing bis-NB-type TCDA, BNBDA, was significantly superior to the bridge-free bis-CH-type H-BPDA in terms of enhancing the *T*_g_. A similar superiority was observed in the comparison of NT-44BP/4,4′-ODA (*T*_g_ = 269 °C [65]) and HTA-44BP/4,4′-ODA (*T*_g_ = 228 °C [29]).

Figure 11b shows a comparison of the 5% weight loss temperature (*T*_d_^5^) in a N_2_ atmosphere, which generally represents thermal stability. The *T*_d_^5^ values decreased in the following order: H-BTA ≈ BNBDA > OHADA > H-PMDA >> CBDA > H-BPDA. The superiority of the H-BTA-based [56] and BNBDA-based systems (#1T) is ascribed to their bridged (bicyclo) structures, which are more resistant to fragmentation for the generation of volatile organic compounds than the bridge-free (mono-cyclo) structure. H-PMDA [56], CBDA [43], and H-BPDA consisting of mono-cyclo structures; in particular, CBDA and H-BPDA provided the PI films with the lowest thermal stability among those of the systems compared here.

Thus, BNBDA was proven to be very effective in enhancing both the *T*_g_ and *T*_d_^5^ (N_2_) of the resultant PI films while maintaining their high optical transparency.

#### 3.6.2. BNBDA-Based PIs Obtained Using Other Aromatic Diamines

The use of BAPP (#2T) as another diamine, despite its high structural flexibility, led to a colorless PI film with an unexpectedly high *T*_g_ close to 300 °C, although a low CTE was not obtained, owing to its poor main-chain linearity. A prominent feature of the PI film was observed in its extremely high toughness (*ε*_b max_ = 124%).

The BNBDA/TFMB system (#5T) afforded a highly transparent, free-standing PI film. However, the resulting brittle film hindered the mechanical testing. The cast film prepared via chemical imidization (#5C) was more brittle, with cracks, owing to poor chain entanglement arising from its insufficient molecular weight. However, it is still possible that the BNBDA/TFMB system can achieve the present targets if the molecular weight is significantly improved. We then attempted to obtain a flexible, free-standing PI film of BNBDA/TFMB by surveying its polymerization routes.

### 3.7. Compatibility of BNBDA-Based Systems to One-Pot Process

#### 3.7.1. Modification of One-Pot Process Conditions

To date, we have reported the effectiveness of the modified one-pot process in enhancing the molecular weights of PIs for systems without sufficient polyaddition reactivity under the condition that their imidized forms are highly soluble [45]. We modified the one-pot process conditions, as briefly mentioned below.

(1) Solvents

Phenolic solvents such as *p*-chlorophenol and *m*-cresol have often been used in one-pot processes [69,70], probably owing to their high dissolution ability and their positive effect on molecular weight enhancement. However, these solvents are unsuitable for large-scale industrial manufacturing because of their toxicity. On the other hand, commonly used amide solvents often cause coloration of the reaction mixture during polycondensation at elevated temperatures, which contributes to the coloration of the resulting PI films. This effect tends to become pronounced when solvents with higher boiling points (e.g., NMP) are used. In the modified one-pot process, *γ*-butyrolactone (GBL), which has a lower dissolution power than phenolic and amide solvents, was selected as an alternative solvent because of its low risk of coloration. GBL also has an advantage during the one-pot process; imidization is easily completed during reflux because of its high boiling point (204 °C). In addition, GBL is superior to amide solvents, owing to its lower hygroscopicity than amide solvents; there is little concern regarding the cloudiness of GBL-based coatings by moisture absorption.

(2) Azeotropic agents

In the conventional one-pot process in solution at elevated temperatures, azeotropic agents (typically benzene, toluene, or xylene) are believed to be indispensable for efficiently eliminating the by-product water during imidization-involving polycondensation to shift the equilibrium to the right. However, the reverse reaction to PAA via the hydrolysis of PI is substantially very slow. Furthermore, the addition of these aromatic hydrocarbons as azeotropic agents, which act as poor solvents for the PIs formed, to the main solvents (in this study, GBL) significantly decreases the total dissolution ability, which is often responsible for the precipitation or gelation of the reaction mixtures. Hence, the modified one-pot process was performed without azeotropic agents. There were no concomitant adverse effects in the absence of azeotropic agents on the molecular weights of the resultant PIs, as is typically observed in the H-PMDA/TFMB system [45].

(3) Temperature-rising profile

The conventional one-pot process is often conducted in two steps: polyaddition at room temperature (first step) and subsequent refluxing at elevated temperatures in solution to complete imidization (second step). In contrast, the modified one-pot process involves the simultaneous progress of amic acid (AA) formation and imidization by prompt heating of the reaction mixtures to 200 °C after the addition of the requisite monomers was completed and holding for 4 h with a continuous N_2_ flow. This rapid heating process was useful for reducing the reaction time and enhancing the molecular weight of the PIs [45].

(4) Catalysts

In the conventional one-pot process, less-volatile aromatic bases such as quinoline (b.p. 238 °C) and isoquinoline (b.p. 242 °C) have often been used as catalysts for the promotion of imidization [71]. In the modified one-pot process, 1-ethylpiperidine (1-EP), which has a much stronger basicity than the above-mentioned aromatic bases, was used as the catalyst. Owing to the higher volatility of 1-EP (b.p. 131 °C), the as-polymerized homogeneous solutions containing 1-EP could be directly used for the subsequent solution casting process without the concerns of residual 1-EP in the resultant film.

Aromatic acids such as *p*-hydroxybenzoic acid and benzoic acid (BA) are also known to act as imidization catalysts [72]. In the modified one-pot process, we found a dramatic effect of the combination of BA and 1-EP on molecular weight enhancement. The amounts and ratios of the catalysts were optimized in this study. After the reaction, BA in the as-polycondensed PI solutions was completely removed by re-precipitation and washing to isolate the PI powder form.

These catalysts were ineffective in enhancing the molecular weights of the PAAs during polyaddition at room temperature, in contrast to their dramatic effect during the one-pot process at elevated temperatures.

(5) Reactors

In the modified one-pot process, we used a well-designed reactor with high sealability based on a magnetic coupling mechanism [45], by which the significant evaporation loss of the volatile catalyst and solvent during reflux at 200 °C can be suppressed.

#### 3.7.2. A Possible Mechanism for the Effect of Prompt Heating on Molecular Weight Enhancement

**Figure 12:**
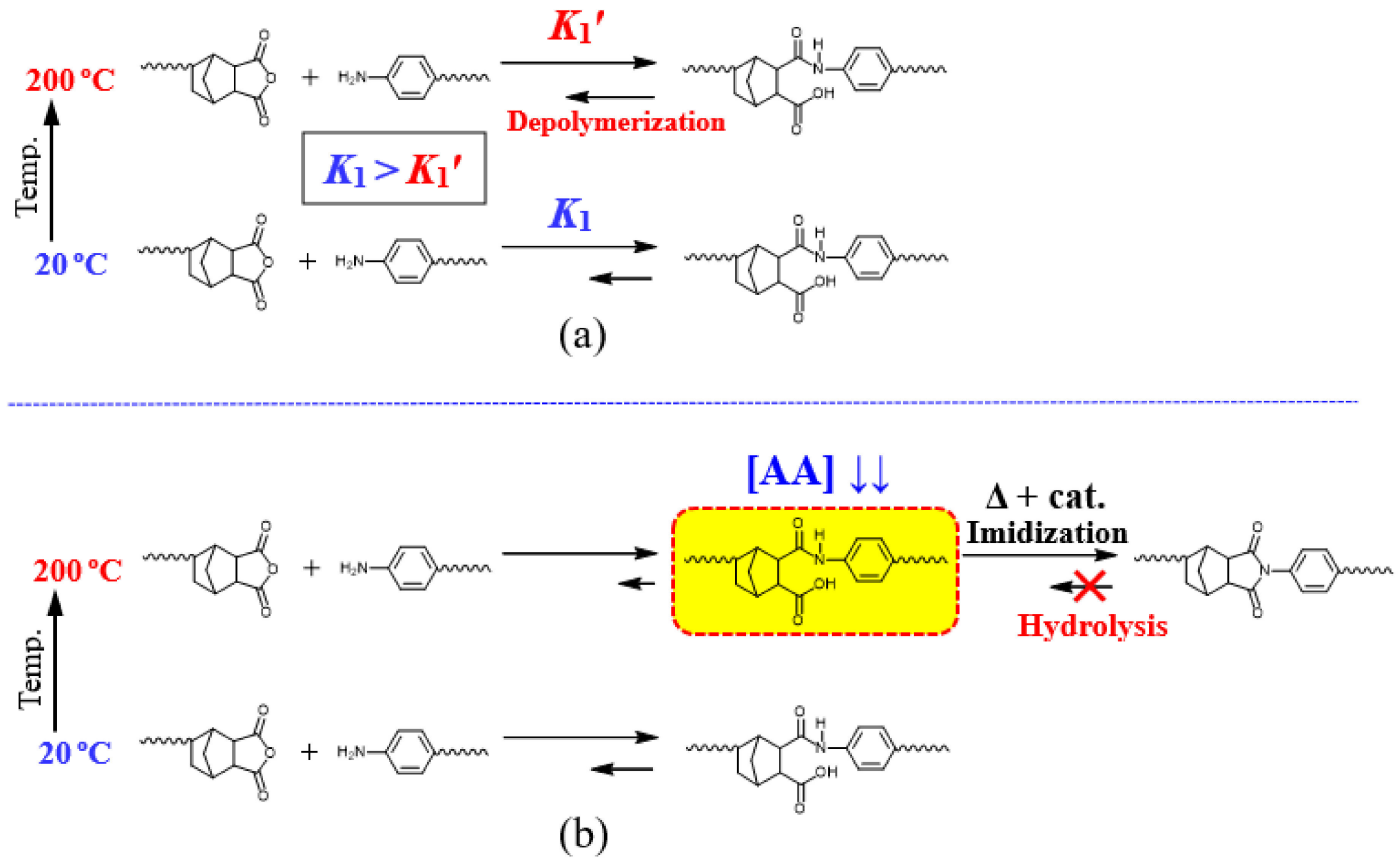
Schematic diagrams for the dependence of equilibrium constants for the addition reaction in solution on temperature: (**a**) case without imidization of the amic acid (AA) units and (**b**) real case including imidization at elevated temperatures (e.g., 200 °C) in solution in the presence of catalysts.

Figure 12 shows the impact of prompt heating of the monomer mixture on the resultant molecular weights of the PIs during the modified one-pot process. In a hypothetical condition where no imidization occurs in solutions even when heated at 200 °C (Figure 12a), based on the fact that the AA formation is essentially an exothermic reaction, the equilibrium constant at 200 °C (*K*_1_′) becomes lower than that at room temperature (*K*_1_). This suggests that partial depolymerization of the PAA very likely occurs in the middle of the temperature-rising process to 200 °C (the above-mentioned second step). It should be true even in the actual systems where imidization also starts to occur from around 150 °C. On the other hand, during prompt heating of the monomer mixtures, AA formation and imidization proceed simultaneously (Figure 12b). In this case, because imide hydrolysis can be virtually regarded as negligible, the AA concentration, [AA], is always kept very low during the rapid heating process (i.e., the lifetime of the AAs is very short). This will cause the equilibrium to shift to the right (i.e., a significant decrease in the functional group concentration, that is, molecular weight enhancement). Thus, reducing [AA] as promptly as possible is the key to molecular weight enhancement.

#### 3.7.3. Individual and Combined Effects of Basic and Acidic Catalysts on Molecular Weight Enhancement and their Proposed Mechanism

**Figure 13:**
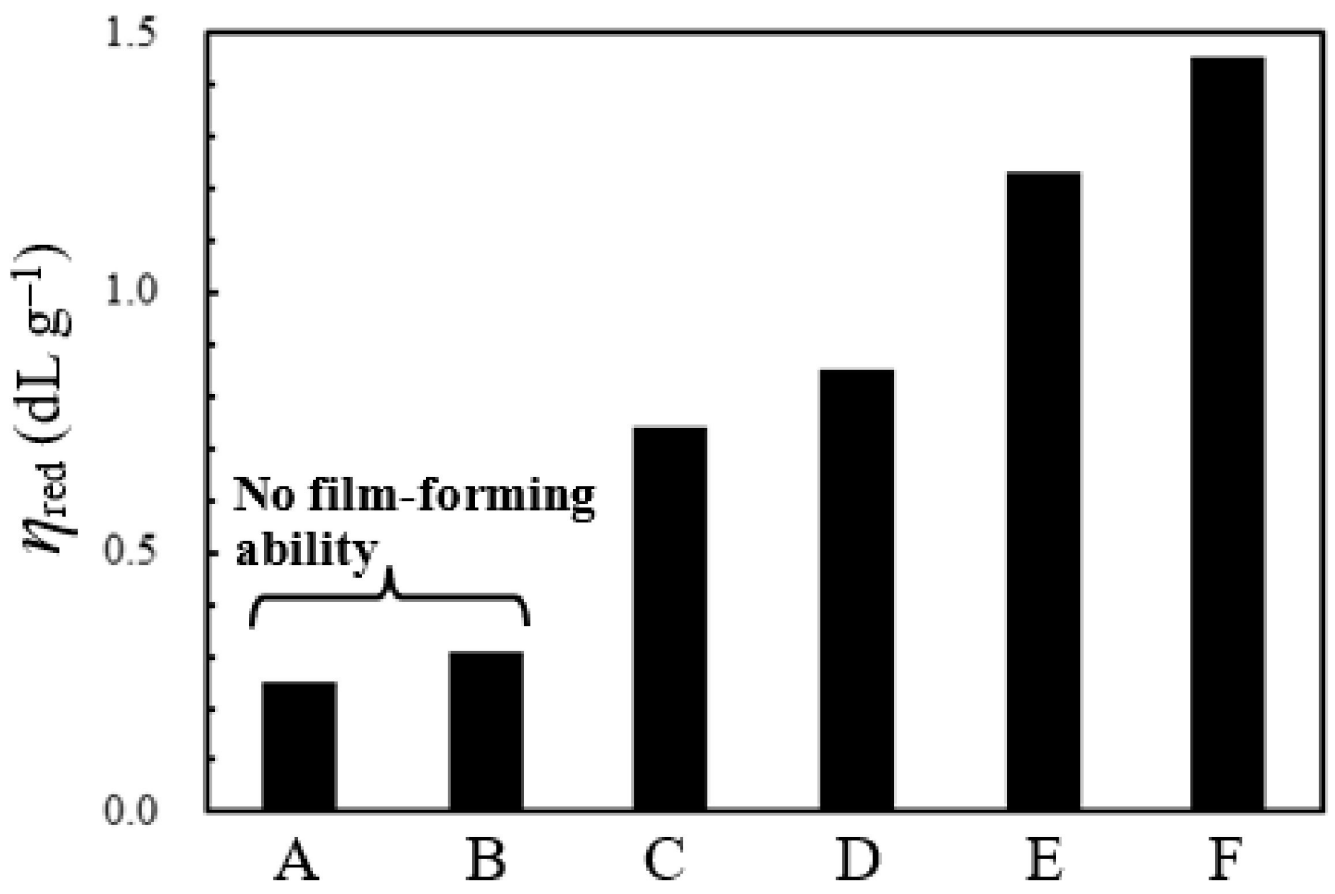
Effects of the polymerization process and catalyst on the *η*_red_ values of the resultant PAA (A) or PIs (B–F) for the H-PMDA/TFMB system. (A): polyaddition at room temperature in DMAc, (B): (A) + chemical imidization, (C–F): one-pot process in GBL, (C): no catalyst, (D): BA (1 equivalent (Eq.)), (E): 1-EP (1 Eq.), and (F): BA (1 Eq.) + 1-EP (1 Eq.).

Imidization catalysts literally have a cyclodehydration (imidization)-promoting effect. On the other hand, they also function concomitantly with the PI molecular weight enhancement, as mentioned in Section 3.7.2. We previously investigated the effects of different polymerization processes (T, C, and R) and the use of 1-EP in the one-pot process on the resultant molecular weights of PIs for the H-PMDA/TFMB system [45]. In addition to our previous results, the effects of BA and the combination of 1-EP and BA are shown in Figure 13.

The polyaddition of H-PMDA and TFMB at room temperature (Figure 13A) led to a very brittle film with cracks, owing to the insufficient molecular weight of the resulting PAA. Chemical imidization (Figure 13B) did not improve film-forming ability. In contrast, one-pot polycondensation without catalysts (Figure 13C) led to a minimum molecular weight required for the formation of a free-standing film. The molecular weight slightly increased with the addition of BA (Figure 13D) during the one-pot process. The impacts of 1-EP (Figure 13E) and the combination of 1-EP and BA (Figure 13F) on molecular weight enhancement were more pronounced. In addition, the molecular weight tended to increase with increasing amount of added 1-EP; however, this was accompanied by gradual increases in the coloration of the solutions and the resulting cast films [45]. Therefore, in this study, the amount of 1-EP was adjusted basically to one equivalent (Eq.), *viz.*, the molar ratio of 1-EP to the theoretical amount of dehydration in the PAAs was equal to unity. Empirically, since the individual use of 1-EP and the combined use of 1-EP and BA were also very effective for other systems, these catalysts were applied to BNBDA-based systems.

**Figure 14:**
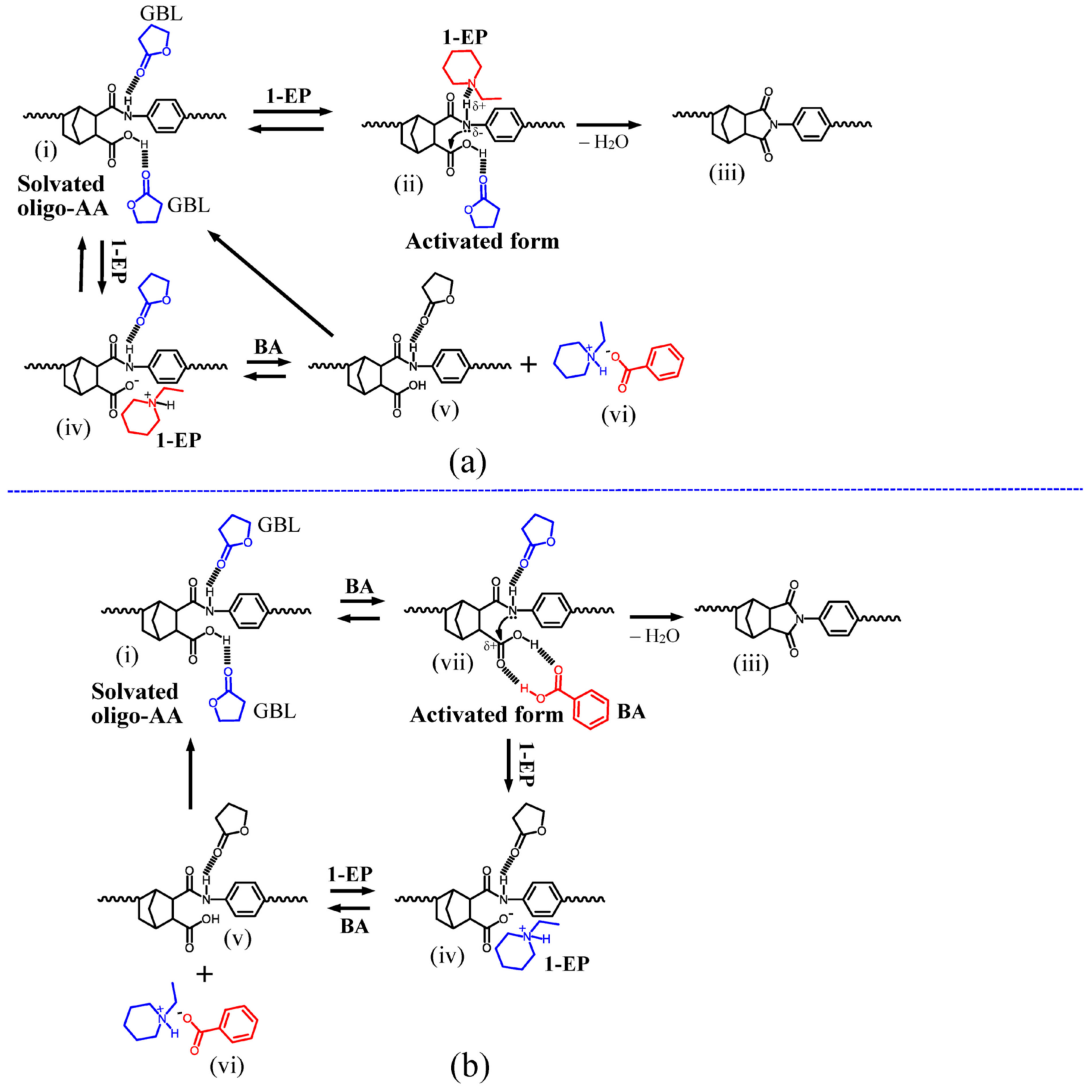
Expected effects of basic (1-ethypiperidine (1-EP)) (**a**) and acidic (benzoic acid (BA)) catalysts (**b**) and their combination during the one-pot process in *γ*-butyrolactone (GBL) at elevated temperatures on the imidization acceleration and the resultant PI molecular weight enhancement. The symbols (i) to (vi) represent expected forms.

The catalytic mechanisms have not been fully discussed in the literature. Figure 14 shows a possible mechanism for the catalytic effects of 1-EP, BA, and their combination. The amic acid oligomers (oligo-AAs) formed in the initial stage of the one-pot process are very likely to be in a solvated state (i) through hydrogen bonding of their COOH and NHCO groups with a large number of surrounding solvent molecules (in this study, GBL) [73,74], as shown in Figure 14a. After the hydrogen-bonded GBL molecules are exchanged for 1-EP, the amide proton is strongly withdrawn from the nitrogen atom of 1-EP; consequently, the nucleophilicity of the NHCO nitrogen atom increases (ii). This promotes its nucleophilic attack on the adjacent C=O carbon atom, resulting in cyclodehydration (iii). In contrast, when the 1-EP molecules form salt bonds with the COOH groups in the AA units (iv), the imidization reactivity is reduced. If BA molecules exist in their neighborhood, as in the 1-EP/BA combined system, the surrounding BA molecules can capture the salt-bonded 1-EP, and, consequently, their imidization reactivity will be recovered in form (v). Simultaneously, a salt is formed between BA and 1-EP (vi), which can be reused as an imidization catalyst.

Figure 14b presents a possible mechanism for the catalytic effect of BA. When BA forms a hydrogen bond with the COOH groups in the AA units (vii), the electrophilicity of the COOH carbon atoms in the AA units increases. This accelerates the imidization. However, when hydrogen-bonded BA is exchanged for 1-EP, which exists in the 1-EP/BA combined system, the AA unit is temporarily converted to a less reactive form (iv) for imidization. However, the surrounding BA molecules can capture the salt-bonded 1-EP; thereby, the AA units can return to their reactive form (v) for imidization. Thus, the proposed mechanism reasonably explains the prominent effects of the individual and combined catalysts.

#### 3.7.4. Polymerizability of BNBDA in Modified One-Pot Process

The results of the modified one-pot polycondensation of the BNBDA-based systems are summarized in Table 9. The BNBDA/4,4′-ODA system (#0R) caused precipitation in the initial stage, and the reaction mixture remained inhomogeneous during the reaction, probably because of the poor solubility of the formed imide oligomer. Unexpectedly, even when another ether-containing diamine with higher structural flexibility, BAPP, was used (#2R), its solution inhomogeneity was not improved.


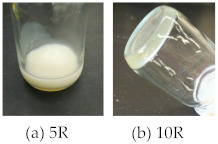


As listed in Table 10 (1), the primary strategy for achieving a low CTE is to ensure the entire main-chain linearity to induce a significant chain alignment parallel to the film plane (*X*–*Y*) direction (called the in-plane orientation). This concept is widely accepted for PI systems obtained via the conventional two-step process [75,76,77,78]. The effectiveness of this structural strategy was confirmed even when PI films were prepared via simple solution casting [20,32,45,55]. Accordingly, a combination of BNBDA and TFMB, both of which are composed of linear/rigid structures, is expected to be the most promising candidate for achieving the present targets.

We previously illustrated that the film preparation route via solution casting (coating, drying, and annealing) from homogeneous PI solutions is superior to that via the two-step process in terms of attaining both higher optical transparency and lower CTEs [55]. In this context, if a stable solution of BNBDA/TFMB polyimide (#5R) with a sufficiently high molecular weight becomes available through the modified one-pot process, the simultaneous achievement of a low CTE and extremely high optical transparency is highly possible by the combined effect of these two strategies (Table 10 (1) and (2)). However, a serious concern arises from the highly linear and rigid main-chain structure of BNBDA/TFMB, that is, a significant decrease in solubility.

Indeed, this concern was correct; the modified one-pot polycondensation of BNBDA and TFMB temporarily maintained solution homogeneity for up to a reaction period of 3 h; however, after that, precipitation occurred, as shown at the bottom of Table 9. This was ascribed to its significantly decreased solubility concomitant with an increase in molecular weight. By contrast, the use of BNBDA analog, H-BPDA (#9R), easily provided a homogeneous solution of the PI with a moderate *η*_red_ value of 0.67 dL g^−1^. This is based on the originally high solubility of H-BPDA/TFMB, which is attributed to its disturbed close chain stacking (aggregation) arising from its highly distorted chain structure, as suggested by the steric structure of H-BPDA (Figure S4a).

To significantly improve the solubility of BNBDA/TFMB while maintaining its chain linearity and rigidity, this system was modified by copolymerization with a small amount of a typical solubility-improving comonomer, 6FDA. One-pot copolymerization with a 6FDA content of 20 mol% (#10R) temporarily led to a homogeneous solution in the initial stage; however, it finally gelled, as shown at the bottom of Table 9. Neither dilution nor heating of the gel was effective for homogenization. In contrast, increasing the 6FDA content to 25 mol% or higher enabled us to obtain homogeneous, viscous, and stable solutions of the PIs. For example, the BNBDA(70);6FDA(30)/TFMB (#12R) copolymer system successfully afforded the PI with a sufficiently high *η*_red_ (1.36 dL g^−1^). In contrast, despite the partial use of 6FDA, the polyaddition of the same copolymer composition (#6) was unexpectedly prohibited by precipitation. This result emphasizes the superiority of the combined approach of controlled copolymerization and modified one-pot process.

### 3.8. Properties of BNBDAbased PI Films Obtained via Modified Onepot Process

#### 3.8.1. BNBDA;6FDA/TFMB Copolymers and Related Systems

Table 11 summarizes the properties of the BNBDA;6FDA/TFMB copolymer films obtained via the modified one-pot process using 1-EP and related systems. The copolymer with 30 mol% of 6FDA (#12R) afforded a non-hazy PI film with excellent optical transparency (*T*_400_ = 83.3% and YI = 2.9), as shown at the bottom of the table, an extremely high *T*_g_ (340 °C by DMA), a relatively low CTE (30.6 ppm K^−1^), a sufficient ductility (*ε*_b max_ = 15.7%), and high thermal stability (*T*_d_^5^(N_2_) = 479 °C). The observed low CTE is closely related to a high extent of in-plane chain orientation induced during solution casting.


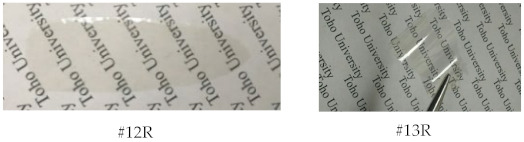


This copolymer, as well as its counterparts with different 6FDA contents, showed excellent solubility in various common organic solvents, including less hygroscopic solvents, such as GBL, cyclopentanone (CPN), and chloroform (Table S1). On the other hand, its H-BPDA-based counterpart (#17R) had similar excellent optical transparency; however, it showed a much lower *T*_g_ (266 °C by DMA), very likely reflecting the allowed internal rotations in the bridge-free CH portions of the H-BPDI units in addition to the disturbed close chain stacking arising from its highly distorted chain structure, which is disadvantageous for the C=O/C=O interactions. Furthermore, the H-BPDA-based copolymer film (#17R) had a much higher CTE (55.9 ppm K^−1^) than the BNBDA-based counterpart (#12R). This indicates that the distorted chain structure of the former disturbed the in-plane orientation behavior during solution casting [45].

**Figure 15:**
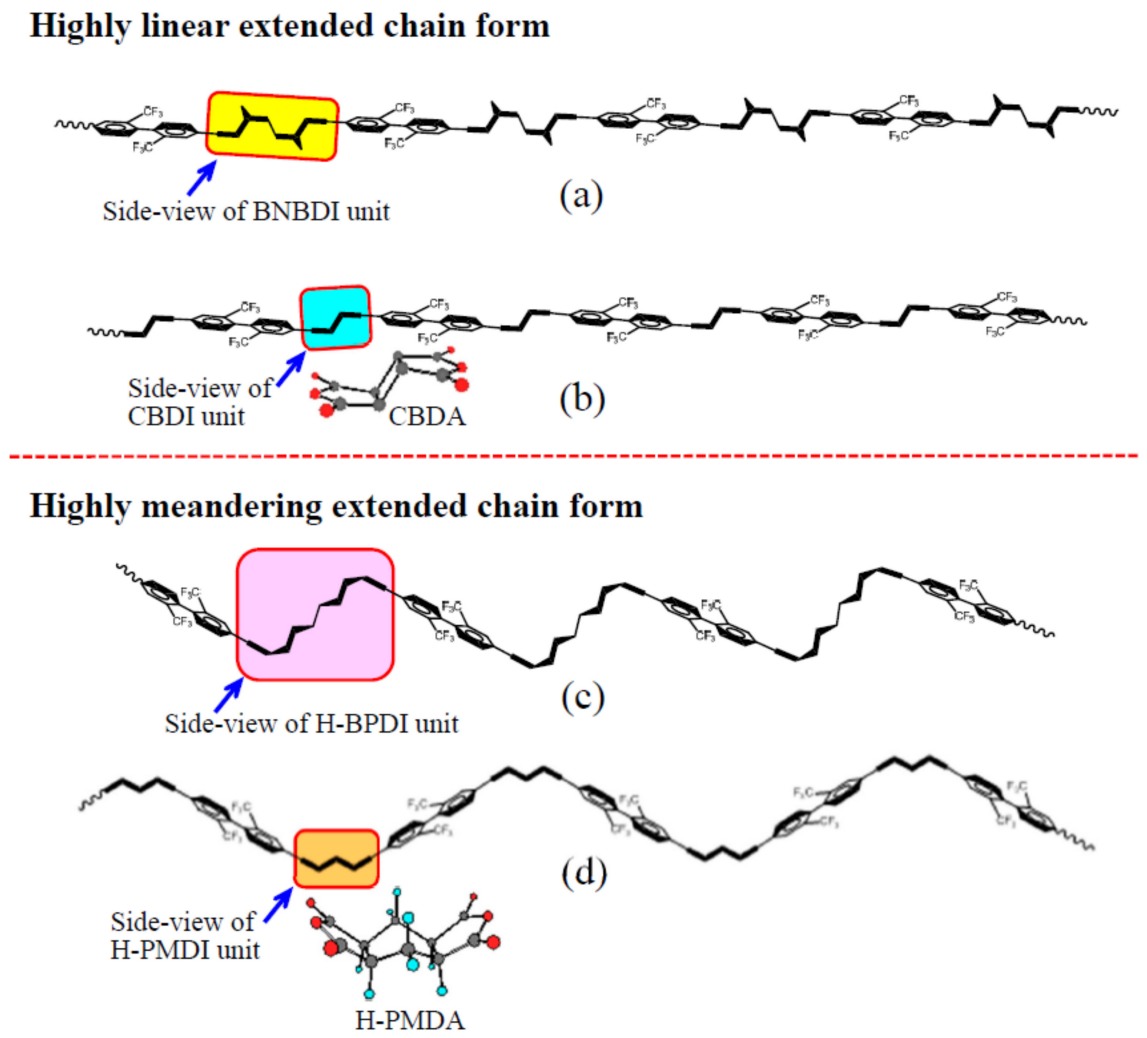
Schematic drawings of highly linear and highly meandering extended chain forms for typical types of TFMB-based PIs: (**a**) BNBDA/TFMB, (**b**) CBDA/TFMB, (**c**) H-BPDA/TFMB, and (**d**) H-PMDA/TFMB systems.

The BNBDA(75)6FDA(25)/TFMB system (#11R) has the highest chain linearity/rigidity among the BNBDA-based systems compatible with the modified one-pot process. This corresponds well to the fact that this copolymer film exhibited the lowest CTE (25.2 ppm K^−1^), although the chain linearity–low CTE relationship remains such a qualitative discussion. Before numerically expressing the chain linearity, we have so far proposed that the degrees of meandering of the schematically drawn extended chains correspond approximately to the measured CTE values of the corresponding actual PI films [31,32,38,44,45,47,59,62]. For example, the CBDA/TFMB system, in which the extended chains can be drawn as shown in Figure 15b, is regarded as having a relatively high overall chain linearity, reflecting a crank-shaft-like steric structure of the CBDI units incorporated into the main chains. Indeed, the actual PI film exhibits a low CTE (21 ppm K^−1^) [31,55].

By contrast, the extended chains of H-PMDA/TFMB inevitably adopt a significantly meandered form, reflecting a distorted steric structure of the H-PMDI units, as shown in Figure 15d. This corresponds to a high CTE value (57 ppm K^−1^) of the actual PI film [45]. A similar situation is observed in the highly distorted H-BPDA/TFMB system (Figure 15c), where the actual film shows a high CTE (57.5 ppm K^−1^, Table 11).

Therefore, the BNBDA/TFMB system, in which the extended chains can adopt a highly linear form, as shown in Figure 15a, is very likely to generate a very low CTE value, aside from the manufacturing problems encountered in this homo PI system. These considerations also suggest that the measured low CTE values of the BNBDA-based copolymers (#11R and #12R) are not “apparent” results arising from some improper experimental conditions mentioned below (specimen defects) but “authentic” results associated with these highly linear main-chain forms.

**Figure 16:**
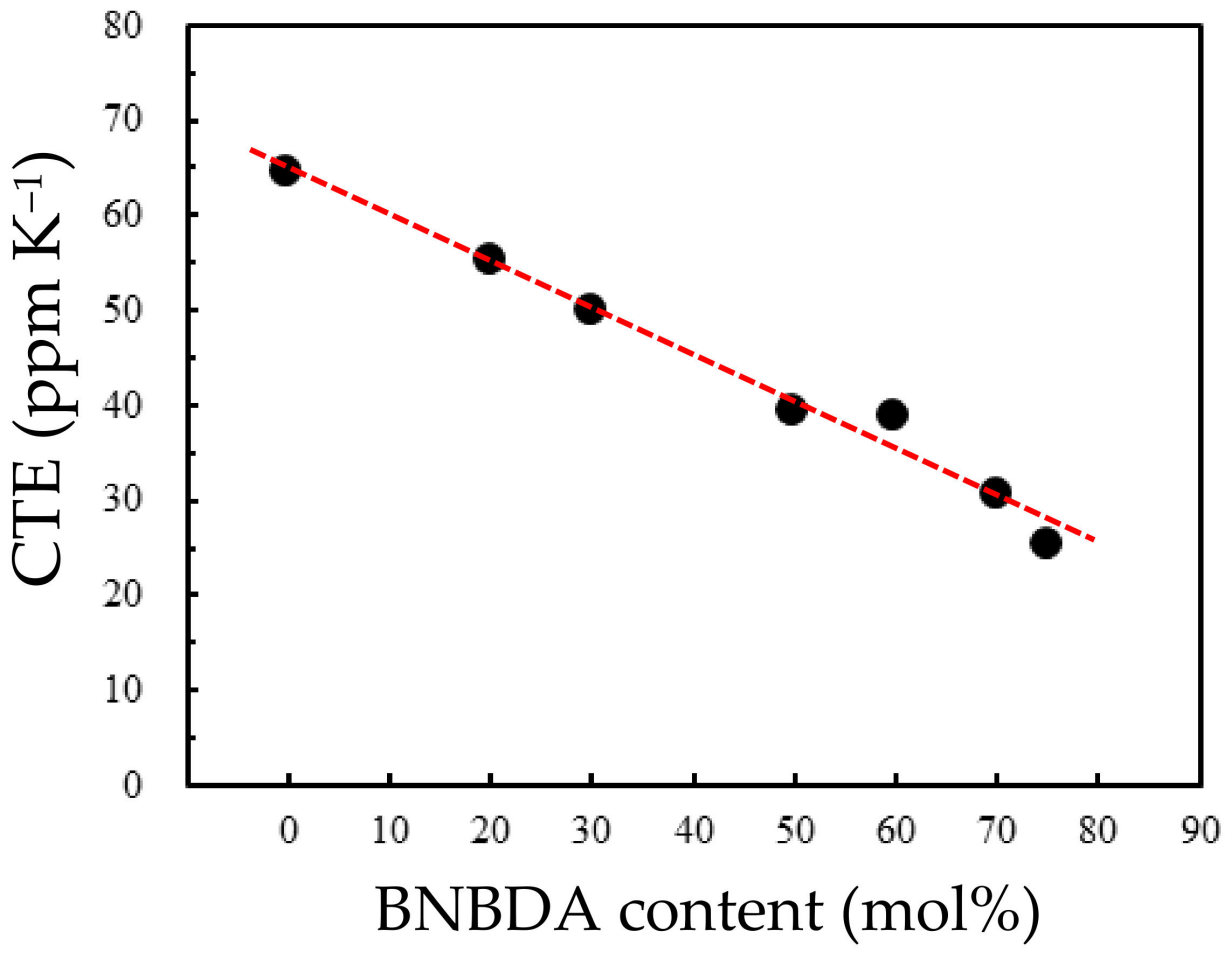
Changes in the CTE with an increase in the BNBDA content in the BNBDA;6FDA/TFMB copolymers.

Table 11 also shows the dependence of the CTE on the BNBDA content of the BNBDA-based copolymers. Reducing the BNBDA content from 75 to 70 mol%, 60, 50, 30, and 20 mol%, the CTE monotonously increased. A good linear relationship between the copolymer composition and CTE (i.e., the additivity rule) was observed (Figure 16), similar to other copolymer systems [43]. If an irreversible film contraction phenomenon during the TMA heating runs, which is ascribed to some possible specimen detects (e.g., relaxation of residual stress, evaporation of residual solvents, and imidization of unimidized portions), affected the CTE values, the additivity rule as shown in Figure 16 would not have been observed.

**Figure 17:**
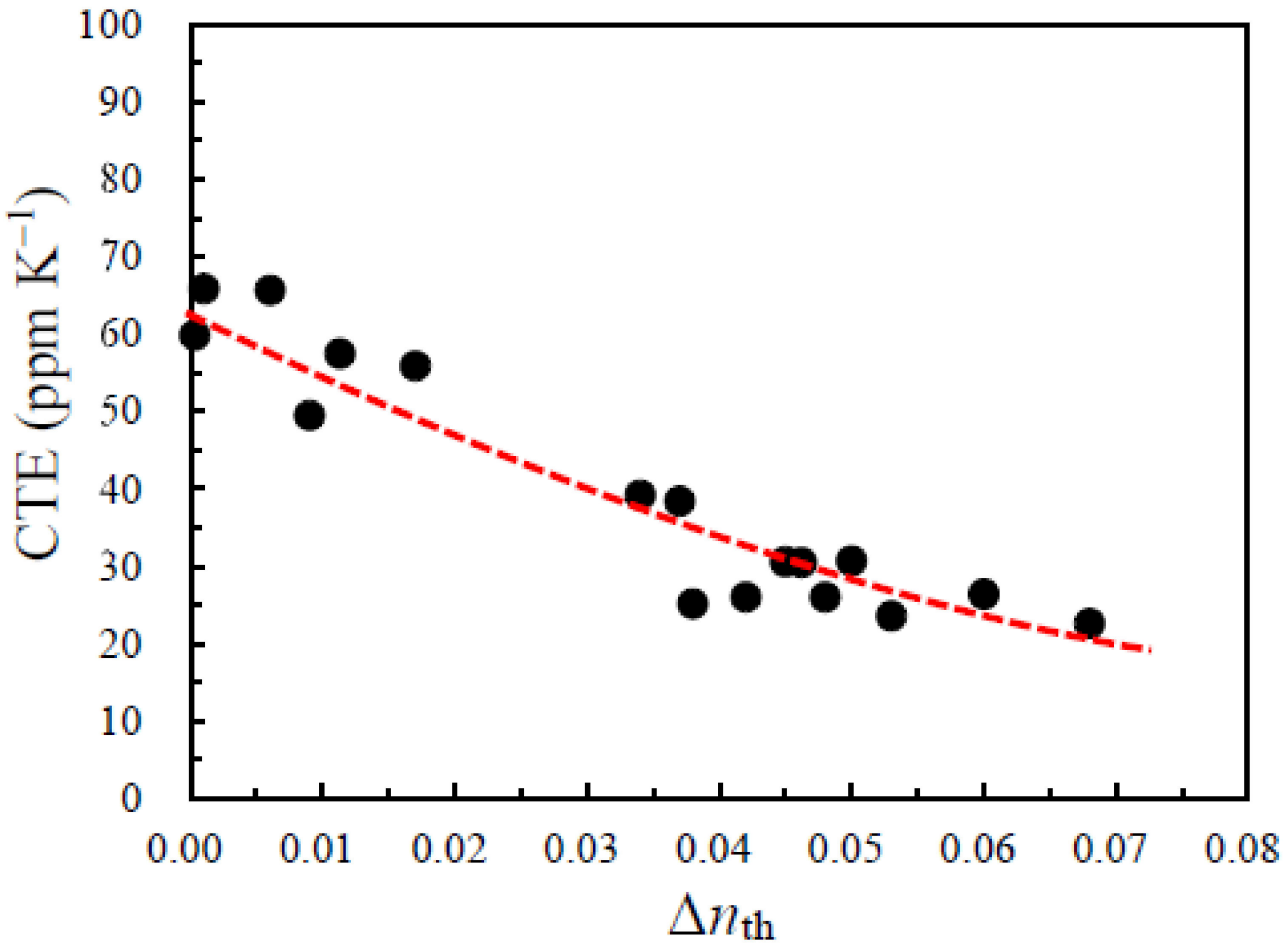
Relationship between the thickness-direction birefringence (Δ*n*_th_) and CTE of the PI films examined in this study.

Figure 17 shows the relationship between the CTE and the thickness-direction birefringence (Δ*n*_th_), which approximately represents the extent of in-plane chain orientation when compared within a similar type of PIs (e.g., semi-aromatic PIs derived from cycloaliphatic TCDAs and aromatic diamines), for the systems examined in this study. A relatively good correlation is observed. This indicates that the measured low CTE values of some BNBDA-based copolymers are ascribed to the in-plane chain orientation and also reconfirms the absence of the adverse impact of the above-mentioned specimen defects on the CTE.

Table 11 also shows a pronounced toughening effect at high 6FDA contents (≥50 mol%) in the BNBDA-based copolymers. This probably results from the significantly improved chain entanglement caused by an increase in the main-chain flexibility due to the introduction of 6FDA with a non-linear distorted structure. An increase in the 6FDA content also improved the thermo-oxidative stability (*T*_d_^5^ (air)), owing to a concomitant decrease in the cycloaliphatic units with lower bond energies.

#### 3.8.2. Impact of the Molecular Weight of PIs on the CTE and Other Properties

We previously found that an increase in the PI molecular weight somewhat contributes to a reduction in the CTEs of PI films obtained via solution casting and proposed its mechanism [32,79]. Based on these findings, the impact of the molecular weight was investigated for BNBDA-based systems, although the effect of this strategy (Table 10 (3)) is empirically much smaller than those of the aforementioned two strategies. Table 12 summarizes the properties of the BNBDA(70);6FDA(30)/TFMB copolymer films (R) with different molecular weights, which were prepared by changing the catalyst conditions based on the results shown in Figure 13. Indeed, the increase in *η*_red_ from 1.36 to 1.96 and 2.86 dL g^−1^ resulted in a gradual decrease in the CTE from 30.6 to 26.0 and 23.6 ppm K^−1^. Thus, hereafter, the combined catalysts were used to maximize the molecular weight of the PIs and, consequently, their CTE-reducing effect.

A gradual increase in *T*_g_ with an increase in *η*_red_ was also observed (Table 12), as generally observed in common polymer systems. This is believed to be related to a decrease in the content of the chain ends, which behave as a structural defect. On the other hand, no clear trend toward film toughening with increasing molecular weight was observed in this case. Thus, there seems to be a limit to further improving the film’s toughness by increasing its molecular weight.

#### 3.8.3. Approach to Improve the Film Toughness while Maintaining Low CTE

##### Modification Using 4,4′- and 3,4′-ODA

As shown in Figure 8, it is difficult in principle to simultaneously achieve a low CTE and high film toughness. Indeed, the BNBDA(75);6FDA(25)/TFMB copolymer (#11R) was not highly tough, although they exhibited a low CTE, as listed in Table 11. In contrast, a significant increase in the 6FDA content (≥50 mol%, #14R and 15R) prominently improved their toughness; however, it was concomitant with an unacceptable increase in the CTE. Then, we used a typical flexible ether-containing diamine, 4,4′-ODA, which can often contribute to an increase in chain entanglement, as the comonomer for modifying the homo BNBDA/TFMB system.

The film properties are listed in Table 13. The modified one-pot copolymerization with a minor content of 4,4′-ODA (20 mol%, #18R) was unsuccessful because of precipitation due to its insufficient solubility, as similarly in the pristine homo system (#5R). The insufficient solubility of this copolymer is plausible if the additivity rule is satisfied for solubility, based on the fact that the BNBDA/TFMB (#5R) and BNBDA/4,4′-ODA systems (#0R) were both incompatible with the one-pot process because of precipitation. Therefore, a further increase in the 4,4′-ODA content would be fruitless for improving the one-pot process compatibility. Instead, 6FDA (30 mol%) was used as an additional comonomer. The modified one-pot process for the copolymer system (#19R) successfully produced a homogeneous solution of the PI with a sufficiently high molecular weight. The resulting copolymer film maintained a relatively low CTE (26.0 ppm K^−1^); however, the toughening effect was not as prominent as expected. The replacement of 4,4′-ODA by 3,4′-ODA (#20R) resulted in a slight decrease in the CTE (24.4 ppm K^−1^) and a slight improvement in the film toughness while maintaining the one-pot process compatibility, as shown in Table 13. However, there is still room for improvement in film toughness.

##### Modification Using BAPP

In our final approach, BAPP, which empirically shows the highest toughening effect, was used as the comonomer to modify BNBDA/TFMB. In addition, a slow drying method (Table 10 (4)) was applied under an established condition (footnote to Table 14) to maximize the total CTE-reducing effect. We previously reported that when PI solutions were coated on a substrate and dried as slowly as possible (initially at lower temperatures), the cast PI films tended to show slightly reduced CTEs [32], although this effect is empirically much smaller than the aforementioned molecular weight effect (Table 10 (3)).


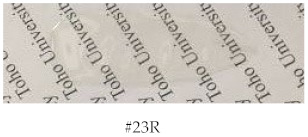


Table 14 summarizes the results of the modified one-pot process and the film properties of the BNBDA/TFMB;BAPP copolymers and related systems. Our initial concern was that the modification of BNBDA/TFMB using BAPP as the comonomer may be disturbed by its one-pot process incompatibility, as predicted by the solubility-related additivity rule based on the fact that the two homo systems (BNBDA/TFMB (#5R) and BNBDA/BAPP (#2R)) were incompatible with the one-pot process because of their poor solubility. Indeed, the copolymerization using 10 mol% BAPP (#21R) was ineffective in obtaining a homogeneous solution. Surprisingly, however, a slight increase in the BAPP content to 20 mol% (#22R) resulted in a homogeneous solution of the PI with a high molecular weight, contrary to the solubility-related additivity rule. An isolated powder sample of this copolymer was highly soluble in various solvents, including some less hygroscopic solvents (Table S1). Solution casting from a homogeneous and stable GBL solution (solid content: ~8 wt%) formed a good-quality PI film. This copolymer (#22R) afforded a highly transparent PI film (*T*_400_ = 81.6%, YI = 3.8, haze = 1.66%). In addition, contrary to our initial concern that the partial use of BAPP, including two ether linkages, may significantly deteriorate its low-CTE and high-*T*_g_ properties, the PI film achieved a low CTE (22.6 ppm K^−1^) and extremely high *T*_g_ (360 °C, by DMA). In addition, it was sufficiently flexible (*ε*_b max_ = 26.8%) with low water uptake (0.43%). The CTE can be reduced a little more by solution casting when using more volatile solvents (typically, CPN) and drying at lower initial temperatures (Table 10 (5)) [62]. However, further systematic investigations using CPN were not conducted in this study because casting from CPN solutions tended to cause slight coloration of the resulting PI films.

Table 14 also shows the impact of the BAPP content of the BNBDA/TFMB;BAPP copolymers on their film properties. A slight increase in the BAPP content from 20 to 30 mol% (#23R) afforded a colorless PI film, as shown at the bottom of this table, with a slight decrease in *T*_g_ and a slight increase in CTE. However, excellent combined properties were still maintained (e.g., *T*_g_ = 340 °C by DMA, CTE = 26.4 ppm K^−1^). In particular, a remarkable toughening effect was observed (*ε*_b max_ = 45.2%) at 30 mol% of BAPP. This copolymer was also superior to its H-BPDA-based counterpart (that is, H-BPDA/TFMB(70):BAPP(30) (#26R)) in terms of higher *T*_g_ and lower CTE, as shown in Table 14.

The increase in the BAPP content from 20 to 30 mol% and 40 to 50 mol% resulted in some inevitable property deteriorations: a gradual decrease in the *T*_g_ and a gradual increase in the CTE, in addition to gradual increases in the *ε*_opt_ and water uptake, which are ascribed to a decrease in the fluorine content.

Polyaddition of the BNBDA/TFMB(50):BAPP(50) system (#7) was disrupted by precipitation (Table 7). Thus, the modified one-pot process is the only method for obtaining a colorless, flexible film of this copolymer.

To date, we have demonstrated that an amide-type fluorinated diamine, AB-TFMB (Scheme 2), is very effective in significantly reducing the CTE while barely maintaining the solubility of the resultant PIs [43,45]. In this study, we attempted to obtain an ultralow CTE using AB-TFMB. However, this approach failed; the one-pot process for the BNBDA/AB-TFMB(50);BAPP(50) copolymer system (#27R) was hindered by precipitation in GBL, similar to DMAc, probably owing to the significantly decreased solubility due to its intensified interchain forces, including hydrogen bonding between the amide groups.

### 3.9. Performance Balance

Practically, it is essential to achieve each target property at higher levels in a well-balanced manner. In this study, the performance balance of some typical BNBDA-based PIs and related systems was overviewed using spider charts prepared based on the criteria for ranking the achievement levels of each target property (Table 15). The five-step ranking was conducted as follows: realistically achievable maximum and minimum levels were ranked 5 and 1, respectively. The intermediate levels were ranked 2–4 by evenly allocating the span between ranks 5 and 1. For example, for the ranking of *T*_g_, *T*_g_s ≥ 360 °C or non-detectable *T*_g_ even by DMA were ranked 5 out of 5, which is based on actual data, that is, *T*_g_ = 360–370 °C [80] for *s*-BPDA/*p*-PDA, which is known as a typical high-*T*_g_ PI. On the other hand, *T*_g_s ≤ 200 °C were ranked 1 out of 5, which was determined with reference to *T*_g_ = 215 °C [69]) for a poly(ether imide) derived from bisphenol A-type TCDA and *m*-PDA, which is a typical low-*T*_g_ PI. The span (360–200 °C) was evenly allocated to an intermediate rank (4–2). Regarding the ranking of CTE, CTEs ≤ 10 ppm K^−1^ were ranked 5 based on CTE = 5–15 ppm K^−1^ [77,78]) of *s*-BPDA/*p*-PDA, known as a typical low-CTE PI, and CTEs ≥ 70 ppm K^−1^ were ranked 1 based on the CTEs of many common polymers (50–70 ppm K^−1^). For the ranking of film toughness, *ε*_b max_ ≥ 80% was ranked 5 based on *ε*_b_ = 85% [54]) for PMDA/4,4′-ODA known as a typical highly tough PI film, and *ε*_b max_ ≤ 2% or none of the film-forming ability was ranked 1. As for the ranking of optical transparency, *T*_400_ ≥ 85% was ranked 5 based on *T*_400_ = 84% [45]) of H-PMDA/TFMB, known as a typical colorless PI, and *T*_400_ ≤ 5% was ranked 1 based on the fact that many wholly aromatic PI films have virtually zero *T*_400_. To rank the solubility, PIs that are highly soluble in less hygroscopic solvents such as GBL and provide stable solutions with high solid contents (≥~10 wt%) were ranked 5, and insoluble PIs in any solvents were ranked 1.

**Figure 18:**
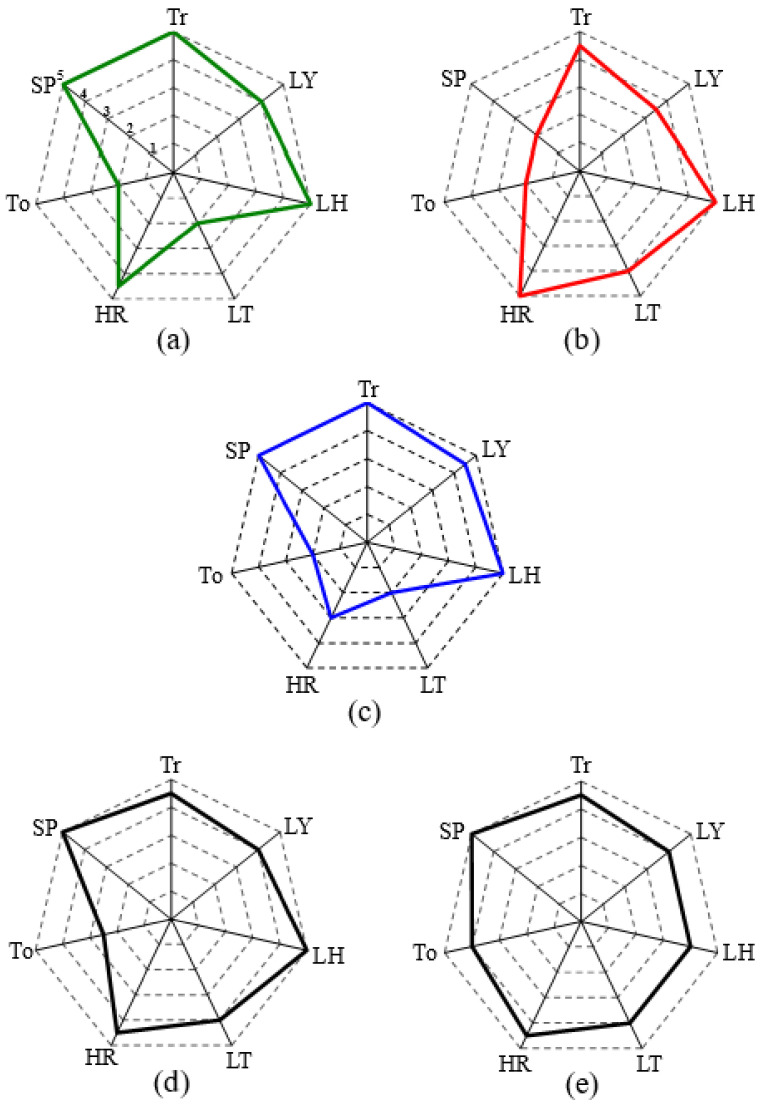
Performance balance of the BNBDA-based low-CTE PIs obtained in this study and related systems: (**a**) H-PMDA/TFMB (R), (**b**) CBDA/TFMB (T), (**c**) H-BPDA/TFMB (R), (**d**) BNBDA(70);6FDA(30)/TFMB (R), and (**e**) BNBDA/TFMB(70);BAPP(30) (R). The criteria and abbreviations of the target properties are shown in Table 15.

Figure 18 shows the spider charts for the selected BNBDA-based PIs and related systems. As is clearly shown from the distorted shape of the chart (Figure 18a), the H-PMDA/TFMB system is disadvantageous in terms of low CTE and suitable toughness. By contrast, the CBDA/TFMB system shows a low CTE, although there are serious drawbacks in terms of film toughness and solubility (Figure 18b). The H-BPDA/TFMB system has a prominent feature, i.e., excellent solubility; however, it is not suitable for achieving low CTE, high *T*_g_, and high toughness (Figure 18c). These three systems are colorless in common, but some important target properties are missing, as evidenced by their biased charts. In contrast, the BNBDA(70);6FDA(30)/TFMB copolymer system has a relatively good performance balance, except for its film toughness (Figure 18d). On the other hand, as shown in Figure 18e, the evenly and well-expanded spider chart for the BNBDA/TFMB(70);BAPP(30) copolymer system indicates an excellent performance balance, that is, its high practicality. Thus, the BNBDA-based PI films developed in this study are promising novel, heat-resistant, colorless polymeric materials suitable for various optoelectronic applications.

## 4. Conclusions

In this study, BNBDA-based colorless PIs with high *T*_g_s, low CTEs, and high film toughness were developed for various optoelectronic applications. Single-crystal X-ray structural analysis of a BNBDA-based model compound suggested that BNBDA has a crank-shaft-like rigid/linear steric structure. BNBDA showed a relatively high polyaddition reactivity with 4,4′-ODA in DMAc at room temperature, leading to a homogeneous solution of PAA with a sufficiently high molecular weight. In contrast, polyaddition with BAPP resulted in precipitation in DMAc, whereas the use of NMP improved the solution homogeneity during the reaction. For the combinations of BNBDA and rigid diamines (*p*-PDA and DABA), polyaddition was unsuccessful because of gelation in both DMAc and NMP, reflecting their rigid chain structures. On the other hand, the reaction mixture of BNBDA and TFMB became barely homogeneous in DMAc after prolonged stirring for 9 days and resulted in PAA with a low *η*_red_ value (0.39 dL g^−1^). Thermal imidization via solution casting of this PAA resulted in a very brittle film, owing to its insufficient molecular weight, similar to that of the film prepared via chemical imidization. Thus, except for the BNBDA/4,4′-ODA system, obtaining flexible, free-standing, BNBDA-based PI films via the conventional two-step process was unsuccessful.

A ductile, free-standing PI film of BNBDA/4,4′-ODA could be obtained by optimizing the thermal conditions of the PAA solution coating, rough drying, thermal imidization on a substrate, and annealing without the substrate. This PI film showed an extremely high *T*_g_ (361 °C), close to that of CBDA/4,4′-ODA, reflecting the inhibited internal rotations by the bridging groups in the NB portions of the BNBDI units. The BNBDA/4,4′-ODA film (T) also exhibited the highest thermal stability (*T*_d_^5^ (N_2_)) compared to its counterparts derived from other related cycloaliphatic TCDAs and 4,4′-ODA, reflecting the bridged (bicyclo) structure in the BNBDI units, which is resistant to fragmentation due to aliphatic C–C bond cleavage at elevated temperatures. The BNBDA/BAPP film (T) also maintained an unexpectedly high *T*_g_ (294 °C) despite the use of highly flexible ether-containing BAPP, with a prominent feature, which is significantly improved film toughness. However, low CTE property was never obtained when these ether-containing diamines were used, owing to the disturbed in-plane chain orientation during the solution casting process. In this paper, the relationship between the degrees of meandering of the schematically drawn extended chains and the CTE values of the corresponding actual PI films was also discussed.

To obtain the flexible BNBDA/TFMB PI film, which is the most promising candidate for achieving the goal of this study, we applied the modified one-pot process in the presence of catalysts. The effects of the temperature-rising profile and catalysts on the resultant molecular weights of the PIs and their mechanisms were discussed. Unfortunately, the modified one-pot process was unsuccessful for the homo BNBDA/TFMB system because of precipitation, reflecting its very rigid/linear chain structure, whereas its H-BPDA-based counterpart produced a homogeneous PI solution via the modified one-pot process, probably owing to its high solubility caused by the highly distorted structure of the H-BPDI units. The homo BNBDA/TFMB system was modified by one-pot copolymerization with 6FDA to improve its solubility. The use of 6FDA (≥25 mol%) solved the problem of one-pot process incompatibility and led to homogeneous solutions of PIs with sufficiently high molecular weights. For example, the BNBDA(70);6FDA(30)/TFMB film exhibited excellent combined properties, including high optical transparency, extremely high *T*_g_, relatively low CTE, and sufficient ductility. The observed low CTE results from a significant in-plane orientation induced by the maintained main-chain linearity. In contrast, its H-BPDA-based counterpart showed a significantly increased CTE and decreased *T*_g_, reflecting its non-linear chain structure based on the highly distorted steric structure of the H-BPDI units. The effect of the PI molecular weight on the CTE was also investigated. The CTE can be further reduced by optimizing the catalyst conditions, consequently enhancing the molecular weight.

The improvement in film toughness while maintaining a low CTE was also challenged, despite the presence of the trade-off between them. The modification of BNBDA/TFMB by copolymerization with BAPP (≥20 mol%) was very effective in ensuring solution homogeneity during the modified one-pot process, deviating from the solubility-related additivity rule, as is evident from the fact that neither homo BNBDA/TFMB nor BNBDA/BAPP was compatible with the one-pot process because of their poor solubility. The BNBDA/TFMB(70);BAPP(30) copolymer successfully achieved the well-balanced properties as the present goal, i.e., significantly improved film toughness (*ε*_b max_ = 45%) while maintaining a low CTE (26.4 ppm K^−1^), extremely high *T*_g_, (340 °C), and excellent optical transparency (*T*_400_ = 83.1%, YI = 2.9, and haze = 1.35%). Thus, the combinations of structural modifications of BNBDA/TFMB, application of the modified one-pot process effective for molecular weight enhancement, and optimization of the film preparation conditions afforded novel colorless PIs that are useful for various optoelectronic applications.

## Figures and Tables

**Table 1:** Abbreviations, commercial sources, and melting points of the common monomers used in this study.

Monomer	Source	Vacuum-Drying Condition	Melting Point ^a^ (°C)
4,4′-Oxydianiline (4,4′-ODA)	Wako Chemical	50 °C/24 h	191
3,4′-Oxydianiline (3,4′-ODA)	Wakayama Seika	50 °C/12 h	79
*p*-Phenylenediamine (*p*-PDA)	Wako Chemical	30 °C/24 h	141
4,4′-Diaminobenzanilide (DABA)	Tokyo Chemical Industry (TCI)	50 °C/12 h	205
2,2′-bis(trifluoromethyl)benzidine (TFMB)	Wakayama Seika	50 °C/12 h	184
2,2-bis[4-(4-aminophenoxy)phenyl]propane (BAPP)	Wakayama Seika	50 °C/12 h	130
4,4′-(Hexafluoroisopropylidene)diphthalic anhydride (6FDA)	Central Glass	160 °C/12 h	248
Dicyclohexyl-3,3′,4,4′-tetracarboxylic dianhydride (H-BPDA)	Iwatani Industrial Gases	160 °C/12 h	212

^a^ Data determined from the endothermic peak temperature measured at a heating rate of 5 °C min^−1^ on DSC.

**Table 2:** Crystal data and structure refinement for AN model.

Empirical formula	C_30_ H_28_ O_4_ N_2_	
Formula weight	480.56	
Temperature	100(2) K	
Wavelength	0.71073 Å	
Crystal system	Monoclinic	
Space group	*P*2**_1_**/c	
Unit cell dimensions	*a* = 15.3504(19) Å	*α* = 90°
	*b* = 6.0664(7) Å	*β* = 98.355(2)°
	*c* = 12.5305(15) Å	*γ* = 90°
Volume	1154.5(2) Å^3^	
*Z*	4	
Density (calculated)	1.382 g/cm^3^	

**Table 3:** Atomic coordinates (×10^4^) and equivalent isotropic displacement parameters (Å^2^ × 10^3^) for AN model. *U*(eq) is defined as one-third of the trace of the orthogonalized *U*^ij^ tensor.

	*x*	*y*	*z*	*U*(eq)
O(2)	6513(1)	3145(2)	7380(1)	27(1)
O(1)	8548(1)	−1328(2)	9294(1)	26(1)
N(1)	7708(1)	1277(2)	8275(1)	19(1)
C(4)	5643(1)	−317(2)	8731(1)	19(1)
C(3)	6416(1)	1272(2)	9093(1)	18(1)
C(2)	7116(1)	−224(2)	9757(1)	18(1)
C(1)	6655(1)	−2494(2)	9720(1)	19(1)
C(6)	5911(1)	−2327(2)	10,421(1)	20(1)
C(5)	5184(1)	−952(2)	9705(1)	19(1)
C(7)	6136(1)	−2468(2)	8568(1)	20(1)
C(9)	6848(1)	2065(2)	8147(1)	20(1)
C(8)	7890(1)	−217(2)	9134(1)	19(1)
C(10)	8297(1)	1718(2)	7509(1)	18(1)
C(11)	8746(1)	3713(2)	7561(1)	22(1)
C(12)	9305(1)	4149(3)	6810(1)	24(1)
C(13)	9409(1)	2613(3)	6019(1)	24(1)
C(14)	8963(1)	626(3)	5975(1)	25(1)
C(15)	8403(1)	161(2)	6724(1)	22(1)
H(4)	5242	174	8095	23
H(3)	6240	2500	9523	21
H(2)	7280	309	10,497	21
H(1)	7051	−3756	9881	22
H(6A)	6113	−1577	11,096	24
H(6B)	5694	−3776	10,576	24
H(5)	4697	−1948	9447	23
H(7A)	6515	−2346	8015	24
H(7B)	5745	−3723	8424	24
H(11)	8673	4741	8091	26
H(12)	9610	5476	6837	29
H(13)	9780	2920	5514	29
H(14)	9037	−399	5444	30
H(15)	8104	−1174	6701	27

**Table 4:** Bond lengths (Å) for AN model.

O(2)-C(9)	1.2137(18)
O(1)-C(8)	1.2072(17)
N(1)-C(9)	1.3918(19)
N(1)-C(8)	1.4045(18)
N(1)-C(10)	1.4364(19)
C(4)-C(7)	1.537(2)
C(4)-C(5)	1.545(2)
C(4)-C(3)	1.545(2)
C(3)-C(9)	1.518(2)
C(3)-C(2)	1.553(2)
C(2)-C(8)	1.512(2)
C(2)-C(1)	1.546(2)
C(1)-C(6)	1.541(2)
C(1)-C(7)	1.543(2)
C(6)-C(5)	1.567(2)
C(5)-C(5)#1	1.524(3)
C(10)-C(11)	1.389(2)
C(10)-C(15)	1.390(2)
C(11)-C(12)	1.387(2)
C(12)-C(13)	1.387(2)
C(13)-C(14)	1.383(2)
C(14)-C(15)	1.390(2)

Symmetry transformations used to generate equivalent atoms: #1 −*x* + 1, −*y*, −*z* + 2.

**Table 5:** Bond angles (°) for AN model.

C(9)-N(1)-C(8)	112.91(12)
C(9)-N(1)-C(10)	122.57(12)
C(8)-N(1)-C(10)	123.97(12)
C(7)-C(4)-C(5)	100.55(11)
C(7)-C(4)-C(3)	101.35(11)
C(5)-C(4)-C(3)	109.99(12)
C(9)-C(3)-C(4)	111.83(12)
C(9)-C(3)-C(2)	104.89(12)
C(4)-C(3)-C(2)	103.59(11)
C(8)-C(2)-C(1)	112.19(12)
C(8)-C(2)-C(3)	104.85(11)
C(1)-C(2)-C(3)	103.05(11)
C(6)-C(1)-C(7)	102.04(12)
C(6)-C(1)-C(2)	107.36(12)
C(7)-C(1)-C(2)	101.02(11)
C(1)-C(6)-C(5)	103.50(11)
C(5)#1-C(5)-C(4)	116.01(15)
C(5)#1-C(5)-C(6)	114.23(15)
C(4)-C(5)-C(6)	102.51(12)
C(4)-C(7)-C(1)	94.68(11)
O(2)-C(9)-N(1)	124.15(14)
O(2)-C(9)-C(3)	127.40(14)
N(1)-C(9)-C(3)	108.41(12)
O(1)-C(8)-N(1)	123.73(14)
O(1)-C(8)-C(2)	127.91(13)
N(1)-C(8)-C(2)	108.31(12)
C(11)-C(10)-C(15)	121.13(14)
C(11)-C(10)-N(1)	119.15(13)
C(15)-C(10)-N(1)	119.72(13)
C(12)-C(11)-C(10)	119.03(14)
C(13)-C(12)-C(11)	120.24(14)
C(14)-C(13)-C(12)	120.38(15)
C(13)-C(14)-C(15)	120.07(15)
C(10)-C(15)-C(14)	119.15(14)

Symmetry transformations used to generate equivalent atoms: #1 −*x* + 1, −*y*, −*z* + 2.

**Table 6:** Torsion angles (°) for AN model.

C(7)-C(4)-C(3)-C(9)	78.17(14)
C(5)-C(4)-C(3)-C(9)	−176.07(11)
C(7)-C(4)-C(3)-C(2)	−34.27(14)
C(5)-C(4)-C(3)-C(2)	71.49(14)
C(9)-C(3)-C(2)-C(8)	−1.06(14)
C(4)-C(3)-C(2)-C(8)	116.34(12)
C(9)-C(3)-C(2)-C(1)	−118.61(12)
C(4)-C(3)-C(2)-C(1)	−1.22(14)
C(8)-C(2)-C(1)-C(6)	177.39(11)
C(3)-C(2)-C(1)-C(6)	−70.36(13)
C(8)-C(2)-C(1)-C(7)	−76.14(14)
C(3)-C(2)-C(1)-C(7)	36.11(14)
C(7)-C(1)-C(6)-C(5)	−30.81(13)
C(2)-C(1)-C(6)-C(5)	74.94(13)
C(7)-C(4)-C(5)-C(5)#1	164.77(16)
C(3)-C(4)-C(5)-C(5)#1	58.47(19)
C(7)-C(4)-C(5)-C(6)	39.60(13)
C(3)-C(4)-C(5)-C(6)	−66.70(14)
C(1)-C(6)-C(5)-C(5)#1	−131.57(16)
C(1)-C(6)-C(5)-C(4)	−5.24(14)
C(5)-C(4)-C(7)-C(1)	−57.64(12)
C(3)-C(4)-C(7)-C(1)	55.44(13)
C(6)-C(1)-C(7)-C(4)	54.36(12)
C(2)-C(1)-C(7)-C(4)	−56.27(13)
C(8)-N(1)-C(9)-O(2)	−170.23(14)
C(10)-N(1)-C(9)-O(2)	1.5(2)
C(8)-N(1)-C(9)-C(3)	7.74(17)
C(10)-N(1)-C(9)-C(3)	179.47(12)
C(4)-C(3)-C(9)-O(2)	62.5(2)
C(2)-C(3)-C(9)-O(2)	174.13(15)
C(4)-C(3)-C(9)-N(1)	−115.37(13)
C(2)-C(3)-C(9)-N(1)	−3.75(15)
C(9)-N(1)-C(8)-O(1)	169.24(14)
C(10)-N(1)-C(8)-O(1)	−2.4(2)
C(9)-N(1)-C(8)-C(2)	−8.47(16)
C(10)-N(1)-C(8)-C(2)	179.94(13)
C(1)-C(2)-C(8)-O(1)	−61.0(2)
C(3)-C(2)-C(8)-O(1)	−172.09(15)
C(1)-C(2)-C(8)-N(1)	116.62(13)
C(3)-C(2)-C(8)-N(1)	5.49(15)
C(9)-N(1)-C(10)-C(11)	82.15(18)
C(8)-N(1)-C(10)-C(11)	−107.04(16)
C(9)-N(1)-C(10)-C(15)	−97.24(17)
C(8)-N(1)-C(10)-C(15)	73.57(19)
C(15)-C(10)-C(11)-C(12)	0.3(2)
N(1)-C(10)-C(11)-C(12)	−179.04(13)
C(10)-C(11)-C(12)-C(13)	0.2(2)
C(11)-C(12)-C(13)-C(14)	−0.5(2)
C(12)-C(13)-C(14)-C(15)	0.3(2)
C(11)-C(10)-C(15)-C(14)	−0.6(2)
N(1)-C(10)-C(15)-C(14)	178.79(13)
C(13)-C(14)-C(15)-C(10)	0.3(2)

Symmetry transformations used to generate equivalent atoms: #1 −*x* + 1, -*y*, −*z* + 2.

**Table 7:** Results of polyaddition of BNBDA with various aromatic diamines and a related system.

No.	TCDA	Diamine	Solvent	Solid Content (wt%) Initial → Final	Reaction Period (Day)	Appearance of Final Reaction Mixture	*η*_red_ (PAA) (dL g^−1^)
0 ^a^	BNBDA	4,4′-ODA	DMAc	30 → 22.2	3	Homogeneous	0.79
1	ibid	ibid	DMAc	30 → 19.6	3	Homogeneous	0.85 (1.05 ^b^)
2	ibid	BAPP	DMAc	30 → 14.0	3	Inhomogeneous (Precipitation)	-
2′	ibid	ibid	NMP	30 → 21.1	3	Homogeneous	0.69
3	ibid	*p*-PDA	DMAc	30 → 8.4	3	Gelation	-
3′	ibid	ibid	NMP	30 → 11.6	3	Gelation	-
4	ibid	DABA	DMAc	30	3	Gelation	-
4′	ibid	ibid	NMP	30	3	Gelation	-
5	ibid	TFMB	DMAc	30 → 19	9	Homogeneous	0.39
6	BNBDA (70)	ibid	DMAc	30 → 10	7	Inhomogeneous (Precipitation)	-
	6FDA (30)						
7	BNBDA	TFMB (50)	DMAc	30 → 16.4	9	Inhomogeneous (Precipitation)	-
		BAPP (50)					
8	H-BPDA	4,4′-ODA	DMAc	30 → 11.3	3	Homogeneous	2.99

^a^ BNBDA without additional purification by sublimation was used; ^b^ maximum value.

**Table 8:** Film properties of BNBDA-based homo PIs and a related system.

No.	TCDA	Diamine	Route	*η*_red_ (dL g^−1^)	*T*_400_ (%)	YI	*λ*_cut_ (nm)	*T*_tot_ (%)	Haze (%)	Δ*n*_th_	*T*_g_ (°C)	CTE (ppm K^−1^)	*E* (GPa)	*ε*_b_ ave/max (%)	*σ*_b_ (GPa)	*T*_d_^5^ (N_2_) (°C)	*T*_d_^5^ (air) (°C)
0T ^a^	BNBDA	4,4′-ODA	T	0.79 (PAA)	84.2	2.0	288	89.2	1.25	0.009	- ^b^	- ^b^	- ^b^	- ^b^	- ^b^	467	404
				(DMAc)													
0C ^a^	ibid	4,4′-ODA	C	0.79 (PAA)	Properties unevaluable due to gelation of the CPN solution												
				0.13 (PI)													
1T	ibid	4,4′-ODA	T	0.85 (PAA)	87.1	1.4	293	89.2	0.63	0.009	361 ^c^	49.5	2.48	10.4/18.5	0.079	498	423
				(DMAc)							384 ^d^						
2′T	ibid	BAPP	T	0.69	81.6	2.3	294	89.2	0.73	0.001	294 ^c^	65.9	2.17	77/124	0.083	477	409
				(NMP)							298 ^d^						
5T	ibid	TFMB	T	0.39 (PAA)	87.0	1.5	287	90.5	0.97	0.007	- ^b^	- ^b^	- ^b^	- ^b^	- ^b^	412	414
				(DMAc)													
5C	ibid	TFMB	C	0.13 (PI)	Properties unevaluable due to the absence of film-forming ability												
8T	H-BPDA	4,4′-ODA	T		85.2	1.6	292	90.3	0.67	0.0003	246 ^c^	59.9	2.19	18.8/41.1	0.072	435	366
				2.99							249 ^d^						
				(DMAc)							259 ^e^						
											256 ^f^						

^a^ BNBDA without further purification by sublimation was used; ^b^ Data not available because of their film brittleness; ^c^ Data measured using DMA (5 °C min^−1^, 0.1 Hz); ^d^ Data measured using TMA (5 °C min^−1^); ^e^ Data reported in Ref. [33] (by DSC measured at 10 °C min^−1^) for the PI obtained from *rel*-(1R,1′*S*,3R,3′*S*,4S,4′*R*)-dicyclohexyl-3,3′,4,4′-tetracarboxylic dianhydride (*cis*-DCDA, Figure S4) and 4,4′-ODA; ^f^ Data reported in Ref. [42] (by DMA measured at 3 °C min^−1^ and 1 Hz) for the PI obtained from *cis*-DCDA and 4,4′-ODA.

**Table 9:** Results of modified one-pot polycondensation of BNBDA with various aromatic diamines in GBL and a related system. The appearance of the reaction mixtures after the reactions are shown below the table: (a) #5R and (b) #10R.

No.	TCDA (mol%)	Diamine (mol%)	Monomer Content (wt%) Initial → Final	Catalyst	Azeotropic Agent	Reaction Period (h)	Appearance of Final Reaction Mixture	*η*_red_ (PI) (dL g^−1^)
0R	BNBDA	4,4′-ODA	20.0 → 8.3	1-EP (1 Eq.)	Toluene	3	Precipitation	-
2R	BNBDA	BAPP	39.5 → 7.1	1-EP (1 Eq.)	-	4	Precipitation	-
				+ BA (1 Eq.)				
5R	BNBDA	TFMB	20.0 → 10.4	1-EP (1 Eq.)	-	6	Precipitation	-
9R	H-BPDA	TFMB	50.0 → 23.5	1-EP (3 Eq.)	Toluene	4	Homogeneous	0.67
10R	BNBDA (80)	TFMB	30.0 → 17.4	1-EP (1.5 Eq.)	-	4	Gelation	-
	6FDA (20)							
11R	BNBDA (75)	TFMB	50.0 → 12.5	1-EP (2.5 Eq.)	-	4	Homogeneous	1.05
	6FDA (25)							
12R	BNBDA (70)	TFMB	50.0 → 12.8	1-EP (2.5 Eq.)	-	4	Homogeneous	1.36
	6FDA (30)							
17R	H-BPDA (70)	TFMB	50.0 → 22.5	1-EP (2.5 Eq.)	Toluene	4	Homogeneous	0.50
	6FDA (30)							
19R	BNBDA	TFMB (90)	50.0 → 10	1-EP (1 Eq.)	-	4	Precipitation	-
		BAPP (10)		+ BA (1 Eq.)				
27R	ibid	AB-TFMB (50)	50.0 → 20	1-EP (1 Eq.)	-	4	Precipitation	-
		BAPP (50)		+ BA (1 Eq.)				

1-EP: 1-ethylpiperidine, BA: benzoic acid.

**Table 10:** Strategies for reducing CTE and predicted disadvantages.

		Strategies	Disadvantages
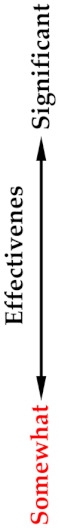	(1)	Main-chain linearity/stiffness ↑	Solubility ↓, film toughness ↓ applicability to polymerization process (T, C, R) ↓
	(2)	Film preparation via solution casting from homogeneous PI solutions	Limited monomers to ensure solubility and low CTE
	(3)	Molecular weights of PIs ↑	Limited polymerization reactivity
	(4)	Slow drying/heating (casting at lower temp.)	Extension of film manufacturing time
	(5)	Solution casting using lower-b.p. solvents	Flammability of solvent vapor ↑

↑ and ↓ represent an increase and decrease in each category.

**Table 11:** Film properties of BNBDA;6FDA/TFMB copolymers and related TFMB-based PIs obtained via modified one-pot process using 1-EP (2.5 Eq.). The photographs at the bottom of the table show the appearance of typical PI films (#12R and #13R).

No.	TCDA (mol%)	*η*_red_ (PI) (dL g^−1^)	*T*_400_ (%)	YI	*λ*_cut_ (nm)	*T*_tot_ (%)	Haze (%)	*ε*_opt_	*T*_g_ (°C)	CTE (ppm K^−1^)	*E* (GPa)	*ε*_b_ ave/max (%)	*σ*_b_ (GPa)	*T*_d_^5^ (N_2_) (°C)	*T*_d_^5^ (air) (°C)
5R	BNBDA	Precipitation after modified one-pot polymerization													
10R	BNBDA (80)	Gelation after modified one-pot polymerization													
	6FDA (20)														
11R	BNBDA (75)	1.05	79.6	4.0	319	89.7	1.37	2.67	330 ^b^	25.2	4.13	5.6/7.5	0.127	482	416
	6FDA (25)								383 ^c^						
12R	BNBDA (70)	1.36	83.3	2.9	317	90.1	0.46	2.65	340 ^b^	30.6	4.84	9.4/15.7	0.139	479	415
	6FDA (30)								374 ^c^						
13R	BNBDA (60)	1.04	83.9	2.7	319	90.2	0.38	2.65	323 ^b^	38.8	4.39	7.9/11.2	0.116	477	420
	6FDA (40)								343 ^c^						
14R	BNBDA (50)	1.14	80.5	4.1	325	90.1	0.46	2.65	333 ^b^	39.2	3.82	24.4/47.6	0.140	482	429
	6FDA (50)								348 ^c^						
15R	BNBDA (30)	1.24	79.4	4.3	331	89.7	0.84	2.65	326 ^b^	50.0	4.31	20.8/51.4	0.137	488	440
	6FDA (70)								327 ^c^						
16R	BNBDA (20)	1.30	79.1	4.7	331	89.5	0.82	2.66	323 ^b^	55.3	-	-	-	496	447
	6FDA (80)								324 ^c^						
9R ^a^	H-BPDA	0.67	88.2	1.5	300	90.8	0.86	2.65	265 ^b^	57.5	3.02	8.3/9.9	0.109	470	419
									274 ^c^						
17R	H-BPDA (70)	0.50	84.5	2.2	330	90.5	0.63	2.65	266 ^b^	55.9	2.77	8.6/12.4	0.109	459	415
	6FDA (30)								275 ^c^						

Thermal conditions for PI film preparation: coating of as-polymerized solutions on a glass substrate, rough drying at 80 °C/2 h in air-convection oven, and heating at 150 °C/0.5 h + 200 °C/0.5 h + 250 °C/1 h on the substrate under vacuum + annealing at 300 °C/1 h without the substrate under vacuum; ^a^ For sample #9R, the annealing process without the substrate was conducted at 240 °C/1 h under vacuum to avoid significant film deformation; ^b^ Data measured using DMA (5 °C min^−1^, 0.1 Hz); ^c^ Data measured using TMA (5 °C min^−1^).

**Table 12:** Film properties of BNBDA(70);6FDA(30)/TFMB copolymers (R) with different molecular weights.

No.	Catalysts (Mol Ratio)	*η*_red_ (PI) (dL g^−1^)	*M*_n_	*T*_400_ (%)	YI	*λ*_cut_ (nm)	*T*_tot_ (%)	Haze (%)	Δ*n*_th_	*ε*_opt_	*T*_g_ (°C)	CTE (ppm K^−1^)	*E* (GPa)	*ε*_b_ ave/max (%)	*σ*_b_ (GPa)	*T*_d_^5^ (N_2_) (°C)	*T*_d_^5^ (air) (°C)
			*M*_w_														
			*M*_w_/*M*_n_														
12R	1-EP	1.36	3.28 × 10^4^	83.3	2.9	317	90.1	0.46	0.043	2.65	340 ^a^	30.6	4.84	9.4/15.7	0.139	479	415
			7.65 × 10^4^														
	(2.5 Eq.)		2.33								374 ^b^						
12R1	1-EP/BA	1.96	4.56 × 10^4^	80.5	4.0	317	89.8	1.11	0.048	2.64	361 ^a^	26.0	4.74	7.6/11.0	0.144	470	436
			1.16 × 10^5^														
	(1/1)		2.54								386 ^b^						
12R2	1-EP/BA	2.86	6.06 × 10^4^	78.6	5.3	313	88.4	1.69	0.053	2.64	366 ^a^	23.6	5.30	8.9/17.4	0.159	471	422
			1.58 × 10^5^														
	(2.5/1)		2.60								384 ^b^						

^a^ Data measured using DMA (5 °C min^−1^, 0.1 Hz); ^b^ Data measured using TMA (5 °C min^−1^).

**Table 13:** Film properties of BNBDA;6FDA/TFMB;ODA copolymers obtained via modified one-pot process using combined catalysts (1-EP + BA).

No.	TCDA	Diamine	*η*_red_ (PI) (dL g^−1^)	*M*_n_	*T*_400_ (%)	YI	*λ*_cut_ (nm)	*T*_tot_ (%)	Haze (%)	*ε*_opt_	*T*_g_ (°C)	CTE (ppm K^−1^)	*E* (GPa)	*ε*_b_ ave/max (%)	*σ*_b_ (GPa)	*T*_d_^5^ (N_2_) (°C)	*T*_d_^5^ (air) (°C)
				*M*_w_													
				*M*_w_/*M*_n_													
5R	BNBDA	TFMB	Precipitation after modified one-pot polymerization														
18R	BNBDA	TFMB (80)	Precipitation after modified one-pot polymerization														
		4,4′-ODA (20)															
19R	BNBDA (70)	TFMB (80)	1.24	3.23 × 10^4^	78.2	2.8	327	89.8	1.03	2.69	321 ^a^	26.0	3.58	5.7/8.5	0.100	479	411
	6FDA (30)	4,4′-ODA (20)		8.16 × 10^4^							376 ^b^						
				2.52													
20R	BNBDA (70)	TFMB (80)	1.65	3.13 × 10^4^	76.5	4.5	320	89.8	0.91	2.68	320 ^a^	24.4	3.61	8.8/15.5	0.117	483	410
	6FDA (30)	3,4′-ODA (20)		9.34 × 10^4^							346 ^b^						
				2.98													

Thermal conditions for PI film preparation: coating of as-polymerized solutions on a glass substrate, rough drying at 80 °C/2 h in air-convection oven, and heating at 150 °C/0.5 h + 200 °C/0.5 h + 250 °C/1 h on the substrate under vacuum + annealing at 300 °C/1 h without the substrate under vacuum; ^a^ Data measured using DMA (5 °C min^−1^, 0.1 Hz); ^b^ Data measured using TMA (5 °C min^−1^).

**Table 14:** Film properties of BNBDA/TFMB;BAPP copolymers and a related system obtained via modified one-pot process using combined catalysts (1-EP + BA). The photograph at the bottom of the table shows the appearance of a typical PI film (#23R).

No.	TCDA	Diamine (mol%)	*η*_red_ (PI) (dL g^−1^)	*T*_400_ (%)	YI	*λ*_cut_ (nm)	*T*_tot_ (%)	Haze (%)	Δ*n*_th_	*ε*_opt_	*T*_g_ (°C)	CTE (ppm K^−1^)	*E* (GPa)	*ε*_b_ ave/max (%)	*σ*_b_ (GPa)	*T*_d_^5^ (N_2_) (°C)	*T*_d_^5^ (air) (°C)	*W*_A_ (%)
5R	BNBDA	TFMB	Precipitation after modified one-pot polymerization															
21R	ibid	TFMB (90)	Precipitation after modified one-pot polymerization															
		BAPP (10)																
22R	ibid	TFMB (80)	5.05	81.6	3.8	293	89.2	1.66	0.068	2.70	360 ^a^	22.6	4.68	14.8/26.8	0.139	491	435	0.43
		BAPP (20)									396 ^b^							
23R	ibid	TFMB (70)	3.18	83.1	2.9	291	89.4	1.35	0.060	2.72	340 ^a^	26.4	4.09	19.2/45.2	0.119	489	427	0.55
		BAPP (30)									362 ^b^							
24R	ibid	TFMB (60)	3.30	83.8	2.6	292	89.4	1.54	0.050	2.74	322 ^a^	30.7	4.16	30.2/61.3	0.125	491	428	0.79
		BAPP (40)									343 ^b^							
25R	ibid	TFMB (50)	3.84	85.2	2.1	291	89.5	1.68	0.046	2.75	319 ^a^	30.5	3.39	34.2/58.3	0.117	487	432	1.02
		BAPP (50)									336 ^b^							
2R	ibid	BAPP	Precipitation after modified one-pot polymerization															
26R	H-BPDA	TFMB (70)	0.86	89.1	0.97	290	90.4	0.60	0.006	2.71	258 ^a^	65.7	1.98	47.2/95.8	0.067	472	437	-
		BAPP (30)									256 ^b^							

Casting condition: GBL solution (solid content: ~8 wt%) was coated on substrates and dried at 65 °C/3 h in an air oven + 150 °C/0.5 h + 200 °C/1 h on substrates under vacuum + 250 °C/1 h without substrates under vacuum; ^a^ Data measured using DMA (5 °C min^−1^, 0.1 Hz); ^b^ Data measured using TMA (5 °C min^−1^).

**Table 15:** Criteria established for ranking the achievement level for each target property.

Properties	Parameters	Ranking				
		1	2	3	4	5
Physical heat resistance (HR)	*T*_g_ (°C)	≤200	220–240	250–270	280–300	≥360 or unclear *T*_g_ by DMA
Low CTE property (LCTE)	CTE (ppm K^−1^)	≥70	60–50	45–35	30–20	≤10
Light Transmittance (Tr)	*T*_400_ (%)	≤5	20–30	40–60	70–80	≥85
Low yellowness (LY)	YI	≥20	10–7	6–4	3–2	≤1
Low haze (LH)	Haze (%)	≥10	4–3	3–2	2–1	≤1
Toughness (To)	*ε*_b max_ (%)	≤2 or no film-forming ability	5–10	20–30	40–60	≥80
Solution processability (SP)	Qualitative solubility	Insoluble	Soluble in amide solvents (≤1 wt%)	Soluble in amide solvents (3–5 wt%)	Soluble in amide solvents (>~10 wt%)	Soluble in non-amide solvents (>~10 wt%)

## Data Availability

The data supporting this study are available within the article and/or in the Supplementary Materials.

## Supplementary Materials

The following supporting information can be downloaded at: https://www.mdpi.com/article/10.3390/polym15183838/s1, Figure S1: Reaction scheme for the synthesis of BNBDA. Figure S2: DSC thermogram of sublimated BNBDA. Figure S3: Thermal behavior of C_12_-model: (a) DSC thermograms in the heating and cooling processes, (b) polarizing optical microscope photograph taken at 135.8 °C during the cooling process from 160 °C above the clearing point (139.6 °C), and (c) expected conformations favorable (i) and unfavorable (ii) for liquid-crystal formation. Figure S4: Steric structures of stereoisomers isolated by Shiotani et al. [33,64]: (a) *rel*-(1R,1′*S*,3R,3′*S*,4S,4′*R*)-dicyclohexyl-3,3′,4,4′-tetracarboxylic dianhydride (*cis*-DCDA) and (b) *rel*-(1R,1′*S*,3R,3′*S*,4R,4′*S*)-dicyclohexyl-3,3′,4,4′-tetracarboxylic dianhydride *(trans*-DCDA). Figure S5: ^1^H-NMR spectrum (DMSO-*d*_6_) of H-BPDA used in this study. Table S1. Results of solubility tests using powder samples obtained via modified one-pot process for BNBDA-based PIs and related systems.
