# Supplementary material for: Colorless Polyimides Derived from 5,5′-bis(2,3-norbornanedicarboxylic anhydride): Strategies to Reduce the Linear Coefficients of Thermal Expansion and Improve the Film Toughness
Source: Polymers (Basel). 2023 Sep 20;15(18):3838. doi: 10.3390/polym15183838 (PMC10535765; doi:10.3390/polym15183838)
Supplement: Supplementary file 1 [file polymers-15-03838-s001.zip › polymers-2604024-supplementary.pdf]

Supplementary Materials

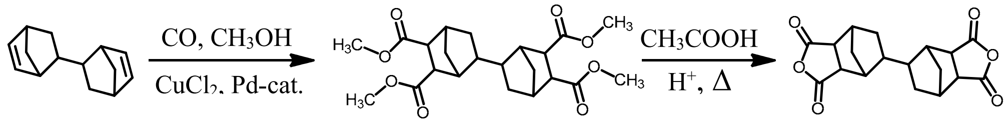

Figure S1. Reaction scheme for the synthesis of BNBDA.

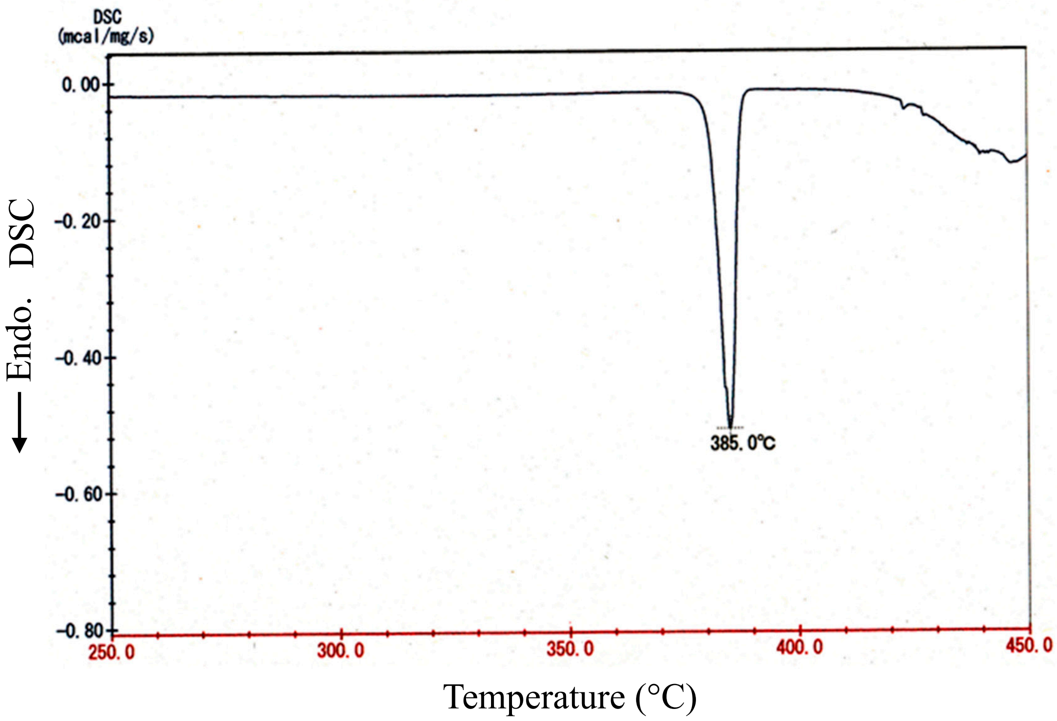

Figure S2. DSC thermogram of sublimated BNBDA.

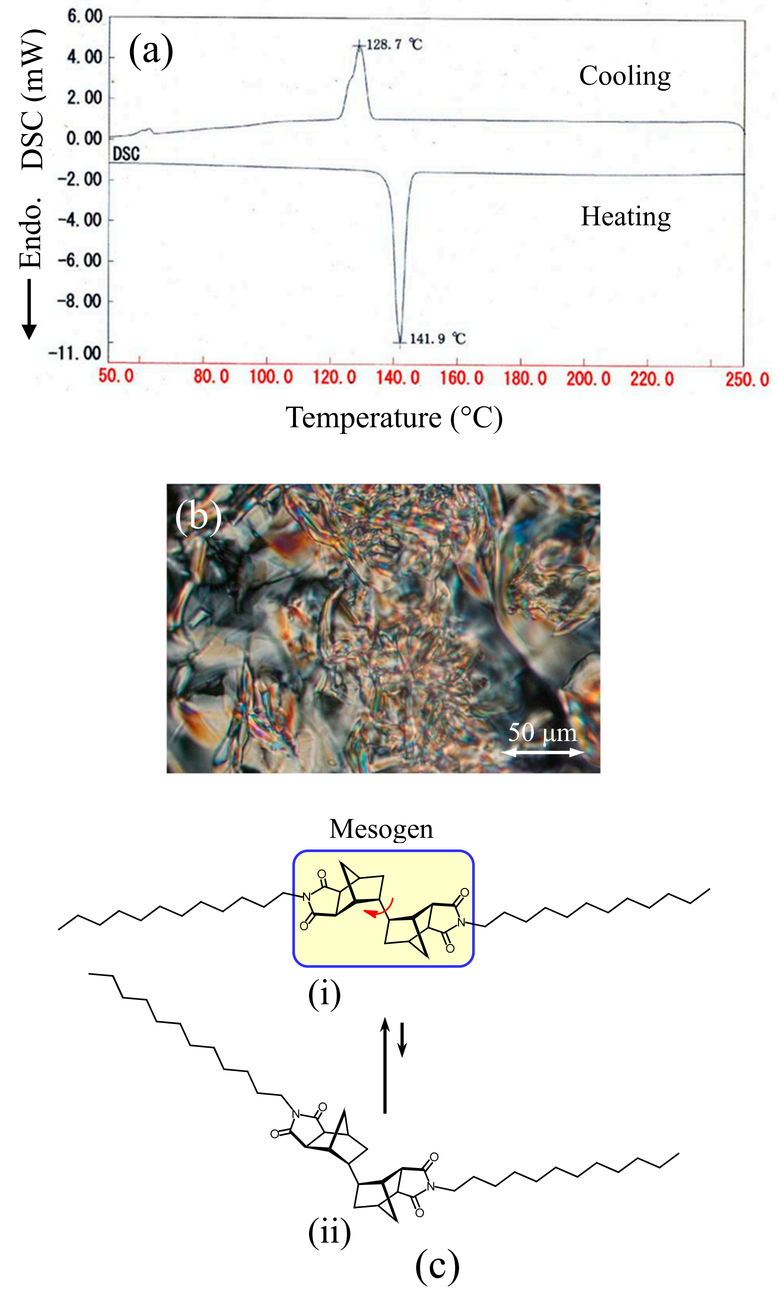

Figure S3. Thermal behavior of C_{12}-model: (a) DSC thermograms in the heating and cooling processes, (b) polarizing optical microscope photograph taken at 135.8 ºC during the cooling process from 160 ºC above the clearing point (139.6 ºC), and (c) expected conformations favorable (i) and unfavorable (ii) for liquid-crystal formation.

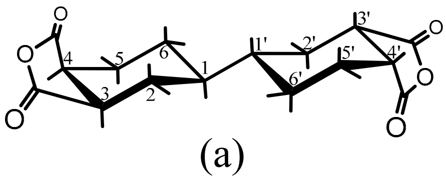

(a)

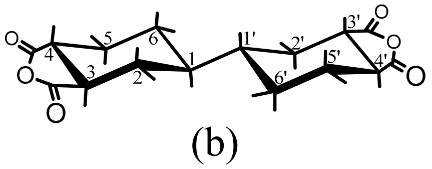

(b)

Figure S4. Steric structures of stereoisomers isolated by Shiotani et al. [**33,64**]: (a) *rel*-(1R,1′*S*,3R,3′*S*,4S,4′*R*)-dicyclohexyl-3,3′,4,4′-tetracarboxylic dianhydride (*cis*-DCDA) and (b) *rel*-(1R,1′*S*,3R,3′*S*,4R,4′*S*)-dicyclohexyl-3,3′,4,4′-tetracarboxylic dianhydride *(trans*-DCDA).

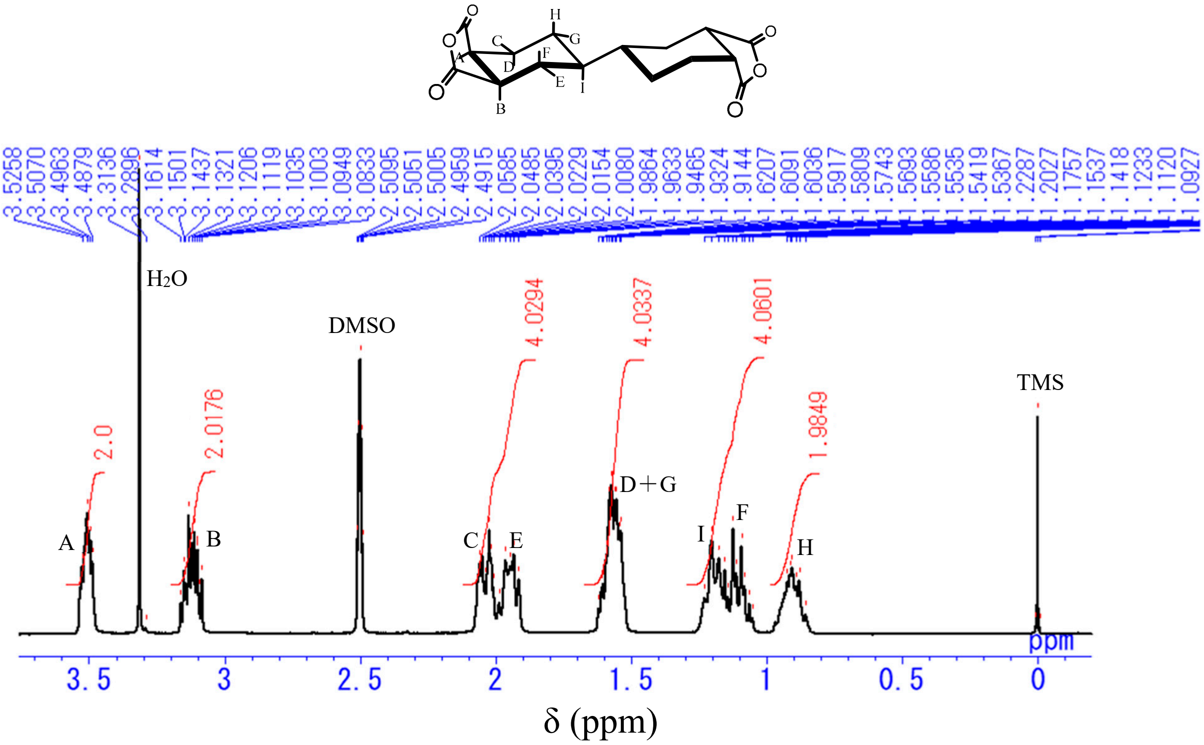

Figure S5. ^{1}H-NMR spectrum (DMSO-d_6) of H-BPDA used in this study.

Table S1. Results of solubility tests using powder samples obtained via modified one-pot process for BNBDA-based PIs and related systems. The appearance of a typical PI powder sample used is shown at the bottom of the table.

No.	TCDA (mol%)	Diamine (mol%)	η_{red} (PI) (dL g^{-1})	C [a] (wt%)	NMP	DMAc	DMF	DMSO	*m*-cresol	CPN	GBL	DOX	THF	CF	ACT	EtAc
					Heating temperature (°C) at the second step											
					150	150	140	150	150	130	150	100	60	50	50	70
12R	BNBDA (70)<br>6FDA (30)	TFMB	1.06	22.5	++	++	++	+	+	++	+	±	+	++	+	–
13R	BNBDA (60)<br>6FDA (40)	TFMB	1.34	16.1	++	++	++	+	+	+	+	±	+	+	±	–
19R	BNBDA (70)<br>6FDA (30)	TFMB (80)<br>4,4′-ODA (20)	1.24	21.7	+	++	++	+	+	++	+	+	+	++	–	–
20R	BNBDA (70)<br>6FDA (30)	TFMB (80)<br>3,4′-ODA (20)	1.65	17.9	+	+	++	+	+	++	+	±	+	+	–	–
22R	BNBDA	TFMB (80)<br>BAPP (20)	5.05	5.5	++	++	++	+	+	+	+	–	±	++	–	–
23R	ibid	TFMB (70)<br>BAPP (30)	3.18	7.5	++	++	++	+	+	++	++	+	+	++	–	–
24R	ibid	TFMB (60)<br>BAPP (40)	3.30	8.2	++	++	++	+	+	++	++	+	+	++	–	–
25R	ibid	TFMB (50)<br>BAPP (50)	3.84	7.9	++	++	++	+	+	++	++	+	+	++	–	–
26R	H-BPDA	TFMB (70)<br>BAPP (30)	0.86	17.5	++	++	++	++	++	++	++	++	++	++	±	±

(++) Soluble at room temperature, (+) soluble when heated at established temperatures, (±) deformed or swelled, and (–) insoluble even by heating.

DMF = *N*,*N*-dimethylformamide, CPN = cyclopentanone, DOX = 1,4-dioxane, THF = tetrahydrofuran, CF = chloroform, ACT = acetone, and EtAc = ethyl acetate.

[a] Solid content in GBL solutions used for solution casting.

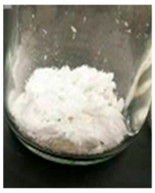

#23R
